# *In Vivo* Molecular Responses of Fast and Slow Muscle Fibers to Lipopolysaccharide in a Teleost Fish, the Rainbow Trout (*Oncorhynchus mykiss*)

**DOI:** 10.3390/biology4010067

**Published:** 2015-02-04

**Authors:** Leonardo J. Magnoni, Nerea Roher, Diego Crespo, Aleksei Krasnov, Josep V. Planas

**Affiliations:** 1 Departament de Fisiologia i Immunologia, Facultat de Biologia, Universitat de Barcelona and Institut de Biomedicina de la Universitat de Barcelona (IBUB), Barcelona 08028, Spain; E-Mails: magnoni@alumni.uottawa.ca (L.J.M); dcrespo@ub.edu (D.C.); 2 Institut de Biotecnologia i de Biomedicina-Parc de Recerca UAB, Universitat Autònoma de Barcelona, Bellaterra 08193, Spain; E-Mail: Nerea.Roher@uab.cat; 3 Nofima, Osloveien 1, N-1432 Ås, Norway; E-Mail: Krasnov@nofima.no

**Keywords:** muscle, lipopolysaccharide, transcriptome, proteome, fish

## Abstract

The physiological consequences of the activation of the immune system in skeletal muscle in fish are not completely understood. To study the consequences of the activation of the immune system by bacterial pathogens on skeletal muscle function, we administered lipopolysaccharide (LPS), an active component of Gram-negative bacteria, in rainbow trout and performed transcriptomic and proteomic analyses in skeletal muscle. We examined changes in gene expression in fast and slow skeletal muscle in rainbow trout at 24 and 72 h after LPS treatment (8 mg/kg) by microarray analysis. At the transcriptional level, we observed important changes in metabolic, mitochondrial and structural genes in fast and slow skeletal muscle. In slow skeletal muscle, LPS caused marked changes in the expression of genes related to oxidative phosphorylation, while in fast skeletal muscle LPS administration caused major changes in the expression of genes coding for glycolytic enzymes. We also evaluated the effects of LPS administration on the fast skeletal muscle proteome and identified 14 proteins that were differentially induced in LPS-treated trout, primarily corresponding to glycolytic enzymes. Our results evidence a robust and tissue-specific response of skeletal muscle to an acute inflammatory challenge, affecting energy utilization and possibly growth in rainbow trout.

## 1. Introduction

In mammals, chronic infection may result in negative energy balance and muscle wasting (cachexia) that causes body weight loss [1]. Muscle wasting is characterized by increased proteolysis of myofibrillar proteins that may lead to muscle atrophy and ultimately death [2]. A signature of this state is the development of a systemic inflammatory process characterized by an elevation of circulating cytokines such as tumor necrosis factor α (TNFα), that may act directly on mammalian skeletal muscle cells increasing protein degradation [1,2,3]. Additionally, skeletal muscle also has immunological capabilities and can express relevant surface molecules, cytokines and chemokines under inflammatory conditions [4,5]. It is known that mammalian skeletal muscle cells are able to recognize specific components of the external cell wall of Gram-negative bacteria such as lipopolysaccharide (LPS) as a response mechanism to bacterial pathogens. This innate immune response is based on the recognition of LPS by Toll-like receptors (TLRs) and the subsequent activation of cellular responses [6] that may lead to decreased muscle growth [3] as well as metabolic changes that can cause significant adjustments in carbohydrate, lipid and protein metabolism [7].

In contrast, the consequences of pathogenic infections on skeletal muscle function in non-mammalian vertebrates, such as fish, are not well documented. Interestingly, fish are believed to be less sensitive to the toxic effects of LPS when compared to higher vertebrates due to important differences in TLR-mediated recognition [8,9]. However, LPS is known to be able to induce an important inflammatory response in fish without the development of septic shock [10,11,12]. As in mammals, LPS stimulates TNFα production in rainbow trout cultured macrophages, indicating that LPS activity and the inflammatory response pathways have been relatively well conserved between fish and mammals [13,14]. Given the well-described effects of the LPS-potentiated catabolic processes in the mammalian skeletal muscle leading to muscle wasting [1,2], it is conceivable that exposure to LPS in fish could also have a potential impact on skeletal muscle physiology and whole body homeostasis. This is important not only from a comparative physiological and immunological perspective but also because fish are important for human nutrition and particularly because skeletal muscle is the edible part of fish. It is also important to stress that the fish skeletal muscle, the engine for swimming, plays a fundamental role not only in locomotion, but also in energy homeostasis, due to the fact that this is a large, metabolically active tissue in fish (nearly 90% of the body mass). The metabolic status of skeletal muscle has important consequences for energy homeostasis in the whole organism, because lipids, carbohydrates, and proteins can be mobilized from this tissue and used as metabolic fuels [15]. In fish, skeletal muscle is composed of anatomically separated slow (red) and fast (white) muscle fibers [16]. The fibers forming the slow skeletal muscle are spread like a superficial sheet covering the bulk of fast fibers, representing approximately 10% and 50%, respectively, of body weight in fish. Both types of muscles also differ functionally, particularly in relation to their contractile activity [17]. The fast muscle is typically active during burst or high-speed swimming and depends largely on anaerobic metabolism supported by glycolysis for energy. On the other hand, the slow muscle participates significantly during sustained swimming, and relies in aerobic metabolism obtained from oxidative phosphorylation in its mitochondria-rich fibers. Furthermore, recent transcriptomic analyses have revealed muscle type-specific responses to swimming in rainbow trout and to LPS administration in the gilthead seabream [18,19]. However, the possibility that LPS administration may cause muscle type-specific transcriptomic responses, or the relationship between transcriptomic and proteomic responses to LPS, have not been evaluated in rainbow trout (*Oncorhynchus mykiss*), a fish species of great economic interest. Therefore, the aims of this study were to investigate the effects of *in vivo* administration of LPS on the transcriptome of slow and fast skeletal muscle in rainbow trout and to compare transcriptomic and proteomic responses to LPS in fast skeletal muscle as it represents the edible tissue in fish. Particularly, the evaluation of the effects of an acute LPS administration on slow and fast skeletal muscle may help us understand the early response of these tissues to an immunological challenge and how it could affect muscle metabolism and function and ultimately fish growth.

## 2. Experimental Section

### 2.1. Animals and Treatment

Adult rainbow trout were purchased from a commercial hatchery (Truites del Segre, Lleida, Spain) and maintained under natural photoperiod in tanks with fresh open water circuits and fed with commercial food. For the LPS injection experiments, trout were separated into two groups (six fish in each group). One group was injected intraperitoneally (ip) with LPS (8 mg·kg^−1^; *E. coli* clone 026:B6, Sigma L-8274, Sigma-Aldrich, Alcobendas, Spain) and the other group (controls) was injected with saline solution (0.9% NaCl). Twenty four or 72 h after injection, during which period the fish were not fed, 3 fish from each group were anesthetized in 3-aminobenzoic acid ethyl ester (0.1 g·L^−1^; Sigma-Aldrich) dissolved in fresh water, sacrificed and slow (red) and fast (white;) skeletal muscle was dissected and frozen in liquid N_2_ until use. Both muscle samples were obtained from standarized locations: slow muscle samples were isolated from the midsection of the lateral line of the caudal region and fast muscle samples were isolated from the anterior dorsal musculature (epaxial) near the dorsal fin and care was taken to avoid cross-contaminating the two different muscle types. The experimental protocols used for trout in this study have been reviewed and approved by the Ethics and Animal Welfare Committee of the University of Barcelona, Spain.

### 2.2. Biochemical Parameters

Blood samples (1–2 mL) were obtained by caudal puncture and centrifuged for 4 min at 12,000× *g*. Plasma was immediately flash-frozen in liquid N_2_ and stored at −80 °C for subsequent analyses. Glucose, lactate and triglyceride levels in plasma were measured using commercial kits (Spinreact, Girona, Spain) and muscle glycogen was isolated and measured as previously described [20]. Results were statistically analyzed by Student’s *t*-test and were considered significant at *p* < 0.05.

### 2.3. RNA Extraction and cDNA Synthesis

Total RNA from fast and slow muscle was isolated using Trizol reagent (Life Technologies, Barcelona, Spain), assessed for quality and quantify with a Nanodrop ND-1000 (Thermo Scientific, Alcobendas, Madrid, Spain), treated with a RQ1 DNAse kit (Promega, Barcelona, Spain) and subsequently reverse-transcribed to cDNA using SuperScript III Transcriptase (Life Technologies), oligo (dT) primer and random hexamer primers (Promega), according to the manufacturer’s instructions.

### 2.4. Microarray Analysis

Microarray analyses were performed on muscle samples using a rainbow trout cDNA microarray platform (SFA2.0 immunochip) previously validated and described [21,22] that has been deposited in Gene Expression Omnibus under accession number GPL6154. This microarray platform includes 1818 unique clones each printed in six replicate spots, and is enriched in genes belonging to the following functional categories: immune response (236 genes), cell communication (291 genes), signal transduction (245 genes) and receptor activity (126 genes), apoptosis (120 genes), cell cycle (76 genes), protein catabolism (90 genes) and folding (70 genes), and response to oxidative stress (39 genes). This cDNA microarray platform has been very useful to describe global transcriptomic responses to pathogens as well as LPS *in vivo* and *in vitro* [10,18,23,24,25,26,27].

Equal amounts of total RNA (1 µg) from slow and fast skeletal muscle samples of individual rainbow trout from the control group (*n* = 3) and the LPS-injected group (*n* = 3) and at each sampling point (24 or 72 h), extracted as described above, were pooled and labeled with Cy3-dUTP and Cy5-dUTP (GE Healthcare, Barcelona, Spain) using SuperScript III reverse transcriptase (Life Technologies). The cDNA synthesis reaction was performed at 50 °C for 2 h in a 20 μL reaction volume, followed with RNA degradation with 0.2 M NaOH at 37 °C for 15 min and alkaline neutralization with 0.6 M Hepes. Labeled cDNA was purified with Microcon YM30 (Millipore, Madrid, Spain). We used a dye swap experimental design and each cDNA from a pooled RNA sample was hybridized to two microarrays, with a total of 24 slides employed in this study. For the first slide, test and control cDNA were labeled with Cy5 and Cy3, respectively, and for the second array dye assignment was reversed. The slides were pretreated with 1% BSA, fraction V, 5 × SSC, and 0.1% SDS (30 min at 50 °C), washed with 2× SSC (3 min) and 0.2× SSC (3 min), and hybridized overnight in a cocktail containing 1.3× Denhardt, 3 × SSC 0.3% SDS, 0.67 μg·μL^−1^ polyadenylate, and 1.4 μg·μL^−1^ yeast tRNA. After hybridization, slides were washed at room temperature in 0.5 × SSC and 0.1% SDS for 15 min, 0.5 × SSC and 0.01% SDS for 15 min, and twice in 0.06 × SSC for 2 and 1 min, respectively. All chemicals were from Sigma-Aldrich. Scanning was performed with ScanArray 5000 and images were processed with QuantArray (GSI Luminomics, Munich, Germany). The measurements in spots were filtered by criteria I/B ≥ 3 and (I − B)/(SI + SB) ≥ 0.6, where I and B are the mean signal and background intensities and SI and SB are the standard deviations. Low-quality spots were excluded from analysis and genes that presented with less than three high-quality spots on a slide were discarded. After subtraction of median background from median signal intensities, the expression ratios (ER) were calculated. Locally weighted non-linear regression normalization was performed, first for the whole slide and then for twelve rows and four columns per slide. To assess the differential expression of genes, the normalized log intensity ratios, or difference of the mean log2 ER from zero, were analyzed for every pair of slides with reverse labeling (six spot replicates per gene on each slide, *n* = 12) with Student’s *t*-test (*p* < 0.01). Due to the large number of genes, the statistical significance of over represented functional categories for each type of muscle at the two different times was assessed using the Yates correction to Chi square test (corrected *p* < 0.05). The log2 ER-ranked up- or down-regulated genes were analyzed, interrogating the functional classes of Gene Ontology [28] and compared by the sums of ranked genes (Student’s *t*-test, *p* < 0.05).

### 2.5. Quantitative Real-Time PCR (qPCR)

In order to validate the results obtained from microarray analysis, quantitative real-time PCR (qPCR) analysis was performed in slow and fast skeletal muscle samples at 24 and 72 h after LPS administration. Specific primers were designed using the Genamics Expression software (www.genamics.com). Primer sequences and GenBank accession numbers of the target genes are presented in Table S1. The qPCR reactions contained 10 μL of SYBR GreenER qPCR SuperMix (Life Technologies), 500 nM of forward and reverse primers, 3 μL RNase/DNase-free water and 5 μL of template DNA (cDNA at a 1:25 dilution or plasmid DNA), in a final volume of 20 μL. The reactions were run in a MyiQ Real-Time PCR Detection System (Bio-Rad, Madrid, Spain) using the following protocol: 2 min at 50 °C, 8 min at 95 °C, followed by 40 cycles of 15 s denaturation at 95 °C and 30 s at 55 °C, and a final melting curve of 81 cycles from 55 °C to 95 °C (0.5 °C increments every 10 s). All the samples were run in triplicate and fluorescence was measured at the end of every extension step. Fluorescence readings were used to estimate the Ct values needed to calculate the number of copies of the gene of interest. Changes in expression were analyzed using a previously described quantification method by qPCR [29]. Using this method, values for each sample were expressed as fold change, calculated relative to control group and normalized for each gene against 18S ribosomal RNA as the reference gene. Expression of 18S was not affected by any of the treatments (less than 7% difference in Ct values between control and treatment samples; *p* > 0.05). All the standard curves exhibited correlation coefficients higher than 0.99, and efficiencies were greater than 99%.

### 2.6. Protein Extraction, Separation and Visualization

Approximately 1 g of frozen fast muscle from pooled control or LPS treated trout (*n* = 3) at 24 h post-injection was crushed in a mortar with liquid N_2_. The powder was resuspended in 5 mL of solubilization buffer (8 M urea, 30 mM Tris-HCl pH 7.5, 2% CHAPS, 1 mM EDTA, 1 mM PMSF, 1 μg·mL^−1^ aprotinin and 2.5 μM leupeptin and pepstatin), homogenized with a Polytron and sonicated (5 × 30 s bursts). After homogenization, the cell extract was centrifuged at 100,000× *g* for 60 min at 4 °C. Supernatants were recovered and frozen at −80 °C until use. An aliquot of cell extract was used to determine protein concentration with the Bio-Rad protein assay using BSA as a standard. Protein samples (500 μg) were adjusted to a final volume of 450 μL in rehydration buffer (8 M urea, 0.5% CHAPS, 0.2% DTT) with ampholines (0.5% v/v). Isoelectrofocusing (IEF) was performed in 24 cm IPG strips pH 3–11 non-linear (GE Healthcare). Once IEF was completed the strips were equilibrated first in 10 mL equilibration buffer (2% SDS, 50 mM Tris-HCl pH 8.6, 6 M urea, 30% glycerol (v/v), 0.002% bromophenol blue) plus 100 mg DTT for 15 min under shaking, and secondly in 10 mL equilibration buffer plus 250 mg iodoacetamide for 15 min under shaking. Separation by protein mass was carried out using an EttanDalt electrophoresis unit (GE Healthcare) on homogenous polyacrilamide gels (12.5%) until the front of bromophenol blue dye reached the bottom of the gel. Gels were stained with Coomassie R 350 Stain (PhastGel blue R, GE Healthcare) or Silver stain, scanned with an ImageScanner (GE Healthcare) and analyzed using ImageMaster 2D Elite 3.1 software (GE Healthcare, Little Chalfont, UK; 2000). Qualitative differences in the abundance of differential protein spots were evaluated and these spots were identified by mass spectrometry (see below).

**Table 1:** Validation of microarray results by qPCR for selected genes in response to LPS ^a^.

GeneBank ID	Clone Name	Slow Muscle				Fast Muscle			
		24 h		72 h		24 h		72 h	
		MA	qPCR	MA	qPCR	MA	qPCR	MA	qPCR
CB511095	Glyceraldehyde-3-phosphate dehydrogenase-3			−1.75	−1.20	−1.44	−1.20		
BX074486	Heat shock protein HSP 90-beta-2					1.25	73.51		
CX146261	Myosin light chain 1, skeletal muscle isoform			−3.78	−14.32				
CU069718	Parvalbumin alpha-3	1.82	4.59	−74.54	−592.22	1.99	5.86	2.38	5.54
DY467707	Serine protease-like protein-1			2.23	3.39	−1.34	−1.83		
CA039449	Tropomyosin alpha 3 chain-2			−2.11	−37.79				
ES325822	Troponin I-4, fast skeletal muscle	1.72	3.53	−5.54	−5.90	1.58	3.25	3.76	2.69
CA371001	Very-long-chain acyl-CoA synthetase					1.27	22.32		

^a^ Results obtained by qPCR are expressed as fold change with respect to the control (saline-injected) group, which was set to 1, with each sample performed in triplicate. Microarray results (MA) are expressed as fold change over control.

### 2.7. Protein Identification by Mass Spectrometry (MS)

Protein spots that were found to be differentially abundant were excised from the gel with a scalpel and digested with trypsin (Sequencing grade modified, Promega). Briefly, proteins were washed sequentially with ammonium bicarbonate buffer and destained with 50% acetonitrile (ACN). Proteins were reduced by treatment with 10 mM DTT and alkylated for 30 min with 100 mM iodine acetamide. They were then sequentially washed and digested overnight at 37 °C with 0.27 nM trypsin. Tryptic peptides were extracted from the gel with 10% formic acid and ACN and extracts were pooled and dried in a vacuum centrifuge. Proteins were either analyzed by MALDI-TOF/TOF MS (4700 Proteomics Analyzer, Applied Biosystems, Framingham, MA, USA) or LC-MS/MS (Q-TOF Global, Micromass-Waters, Manchester, UK). In the first case, the digests were redissolved in 5 μL of 0.1% trifluoroacetic acid (TFA) in 50% ACN. Typically, a 0.5 μL aliquot was mixed with the same volume of a matrix solution, 5 mg·mL^−1^ α-ciano-4-hydroxycinnamic acid (Waters, Barcelona, Spain) in 0.1% TFA in 50% ACN, and directly spotted onto a MALDI-plate. MS spectra were acquired and three major peaks were selected for further characterization by MS/MS analysis. Spectra were submitted for database searching in a generic MASCOT format. Some of the tryptic digested peptide samples (spots number 10–12 in Table 1) were analyzed by on-line LC-MS/MS (Cap-LC-nano-ESI-Q-TOF, CapLC, Micromass-Waters). In those cases, samples were re-suspended in 12 μL of 10% formic acid solution and 4 μL were injected for chromatographic separation in a reverse-phase capillary C18 column (75 μm of internal diameter and 15 cm length, PepMap column, LC Packings). The eluted peptides were ionized via coated nano-ES needles (PicoTipTM, New Objective). A capillary voltage of 1800–2200 V was applied together with a cone voltage of 80 V. The collision in the CID (collision-induced dissociation) was 20–35 eV and argon was employed as collision gas. Data were generated in PKL file format and were submitted for database searching in MASCOT server. Proteomic analyses were performed at the Proteomics Platform of the Parc Cientific de Barcelona at the Universitat de Barcelona.

## 3. Results and Discussion

In the present study, we investigated the transcriptomic and proteomic response of skeletal muscle to an acute immunological challenge in order to understand the physiological response of rainbow trout to bacterial pathogens. For this purpose, we simulated the effects of a bacterial infection in rainbow trout by *in vivo* administration of LPS, an activator of the fish innate immune system known to induce an inflammatory response in this species [11,27,30].

### 3.1. Metabolic Effects of LPS Administration in Rainbow Trout

**Figure 1:**
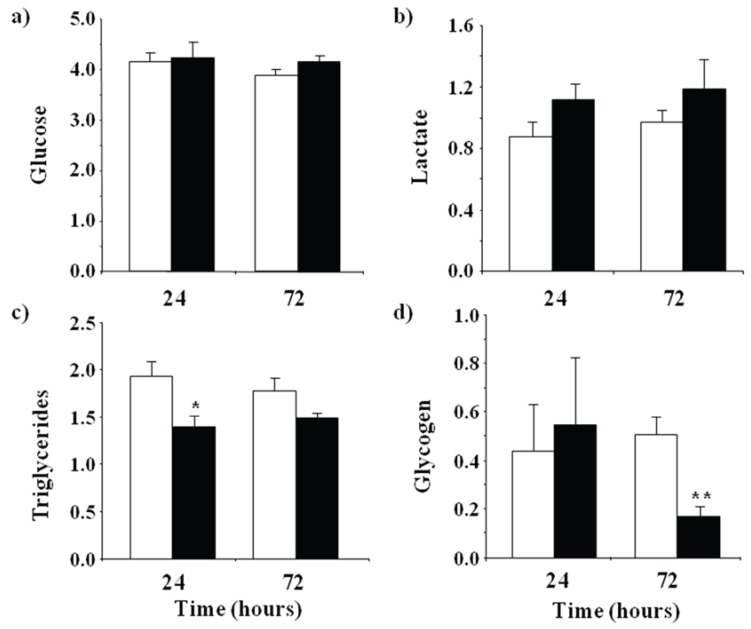
Changes in metabolite levels in plasma and fast skeletal muscle of trout at 24 or 72 h after saline (white bars) or lipopolysaccharide (black bars) injection. (**a**) Glucose; (**b**) lactate and (**c**) triglyceride concentrations in plasma are expressed as mM, where (**d**) glycogen levels in fast muscle are expressed in mg·g^−1^. Values shown are means ± SE of six fish per group, each analyzed in triplicate. Significant differences between control and treatment groups are shown with asterisks (*p* < 0.05).

We first evaluated the metabolic effects of an acute immune challenge on skeletal muscle in rainbow trout by measuring the levels of plasma metabolites (*i.e.*, glucose, lactate and triglycerides) and fast muscle glycogen content at 24 and 72 h after an intraperitoneal injection of LPS or saline. The plasma levels of glucose and lactate were not significantly altered in response to LPS administration at 24 or 72 h post-injection (Figure 1A,B). However, triglyceride plasma levels decreased in LPS-injected fish relative to controls, although only significantly at 24 h (Figure 1C). In addition, glycogen levels in fast skeletal muscle were similar between control and LPS-injected fish at 24 h but were significantly lower in LPS-injected fish at 72 h (Figure 1D). These results suggest that LPS injection in fish may have caused an increase in the utilization of energy sources in the form of circulating triglycerides (lipids) and fast muscle glycogen (carbohydrate) stores. The observed metabolic response to LPS administration in rainbow trout is similar to the consequences of a systemic inflammatory response (sepsis) caused by microbial infections in mammals that are characterized by an increase in the use of glucose by the mammalian host, sometimes followed by a transient hyperglycemia, lactate production, metabolic acidosis and glycogen depletion [31,32]. Therefore, as in mammals, fish may also undergo metabolic changes in response to LPS to likely cover the increased energetic demand caused by an inflammatory insult.

### 3.2. Transcriptomic Analysis of Fast and Slow Skeletal Muscle in Response to LPS Administration in Rainbow Trout

Transcriptomic analysis of trout skeletal muscle was performed using a microarray platform (SFA2.0) previously validated for studies involving response to stress and toxicity [21,22], as well as the immune response in trout [10,11,33]. Our results indicate that LPS administration significantly altered the expression of 131 genes in fast muscle after 24 h (*p* < 0.01), with 59 genes up-regulated and 72 genes down-regulated. At 72 h after LPS administration, 304 differentially expressed genes (DEGs) were found in fast muscle, 156 genes up-regulated and 148 genes down-regulated. In slow muscle, a similar number of DEGs were found at 24 h after LPS administration as in fast muscle: 135 DEGs, with 77 genes up-regulated and 58 genes down-regulated. In contrast, 226 genes were differentially expressed at 72 h in slow muscle, with 120 genes up-regulated and 106 genes down-regulated. Validation of the microarray data was performed by qPCR of eight DEGs for both slow and fast muscle (Table 1). We selected a similar number of genes up- and down-regulated included in several functional categories. In all cases, the direction and magnitude of changes in expression were confirmed by qPCR.

**Figure 2:**
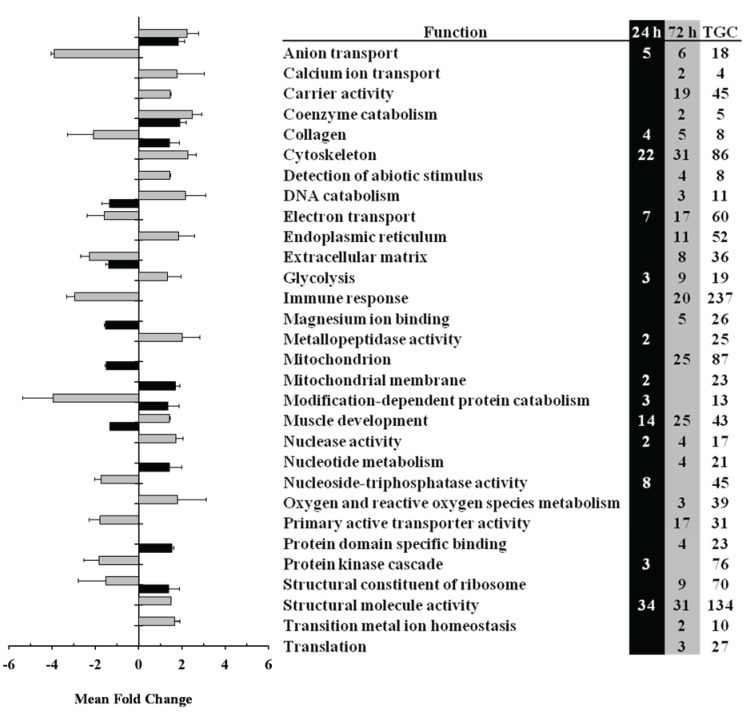
Gene Ontology analysis for slow skeletal muscle at 24 h (black bars) and 72 h (grey bars) after lipopolysaccharide (LPS) administration in trout. Differentially expressed genes (DEGs) were grouped by gene ontology functional categories and fold change differences were analyzed by Student’s *t*-test (*p* < 0.05). TGC indicates the total number of genes included in each functional category. Data are shown as mean fold change.

**Figure 3:**
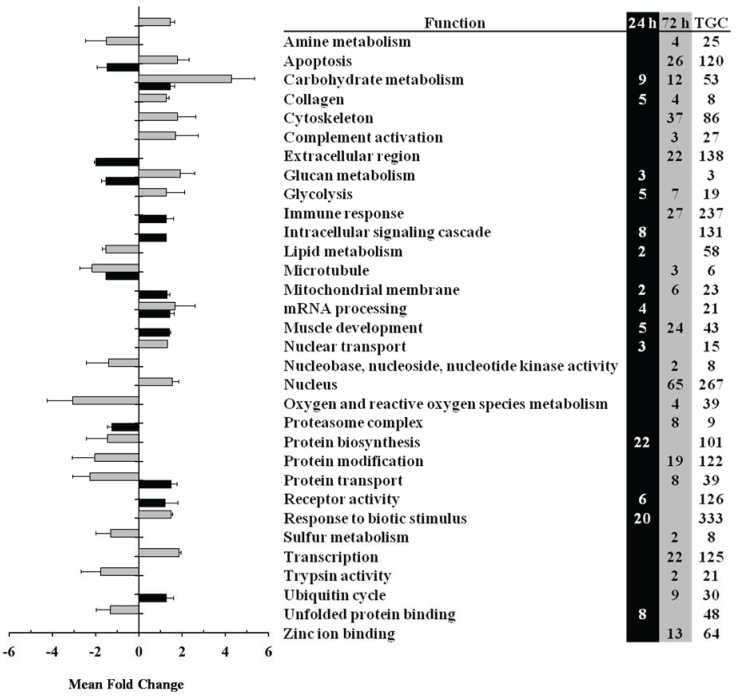
Gene ontology analyses of genes expressed in fast skeletal muscle at 24 h (black bars) and 72 h (grey bars) after lipopolysaccharide (LPS) administration in trout. Differentially expressed genes (DEGs) were grouped by gene ontology functional categories and fold change differences were analyzed by Student’s *t*-test (*p* < 0.05). TGC indicates the total number of genes included in each functional category. Data are shown as mean fold change.

In a first instance, the gene expression data were analyzed by functional categories to evidence overall transcriptomic differences between the two types of skeletal muscle. An initial analysis of over represented Gene Ontology categories provided a first view of the transcriptomic response of slow and fast muscle over time after LPS administration (Figures S1 and S2). This analysis evidenced altered expression of defense and immune responses as well as muscle development and contraction in slow and fast muscle that intensified from 24 to 72 h after LPS administration. The major difference in over represented categories between the two tissues was the significantly altered expression of the categories related to mitochondria only in slow muscle at 72 h after LPS administration (Figure S1). Further analysis identified several functional categories that were significantly (*p* < 0.05) differentially expressed in fast or slow muscle in response to LPS administration (Figure 2 and Figure 3).

In slow muscle, functional categories altered by LPS administration included mitochondrion and electron transport (both up-regulated at 72 h), cytoskeleton, muscle development (up-regulated at 24 h but down-regulated at 72 h), glycolysis (down-regulated at 24 and 72 h) and immune response (up-regulated at 72 h) (Figure 2). In fast muscle, LPS administration caused changes in the expression of functional categories related to carbohydrate metabolism (e.g., glycolyis; down-regulated at 24 h but up-regulated at 72 h), protein metabolism (e.g., protein biosynthesis and modification), muscle development (up-regulated at 24 and 72 h), nuclear activity and transcription, apoptosis and immune response (up-regulated at 72 h) (Figure 3). Within these differentially expressed functional categories, the individual value and direction of change for representative genes are detailed in Table 2 and the full list of DEGs in slow and fast muscle at 24 and 72 h after LPS administration can be found in Tables S2 to S5, respectively.

Within the functional category of carbohydrate metabolism we observed a significant (*p* < 0.05) up-regulation of the mRNA expression levels of genes involved in glycogen breakdown (glycogen phosphorylase-2), glycolysis (beta-enolase and glyceraldehyde-3-phosphate dehydrogenase), and gluconeogenesis (glucose-6-phosphate isomerase) in fast muscle at 72 h after LPS administration that was preceded by a significant down-regulation of most of these genes at 24 h after LPS administration (Table 2, Tables S4 and S5). The mRNA expression levels of 6-phosphofructokinase (6-PFK), a key glycolytic enzyme, were decreased by LPS administration in fast muscle at both 24 and 72 h, suggesting a tight regulation of this metabolic pathway during endotoxin treatment as in the mammalian muscle [34]. In contrast, in slow muscle we observed a marked down-regulation, particularly at 72 h, of DEGs involved in carbohydrate metabolism including several important genes involved in glycolysis (e.g., beta-enolase, glyceraldehyde-3-phosphate dehydrogenase and 6-PFK) (Table 2, Tables S2 and S3).

**Table 2:** Differentially expressed genes representative of various physiological processes in slow and fast skeletal muscle at 24 and 72 h after lipopolysaccharide administration in rainbow trout ^a^.

Clone Name	Slow Muscle		Fast Muscle	
	24 h	72 h	24 h	72 h
**Carbohydrate metabolism**				
Beta enolase-3		−2.85		2.41
6-phosphofructokinase	−1.53		−1.88	
Glyceraldehyde-3-phosphate dehydrogenase-5		−1.84	−1.61	1.66
Glycogen phosphorylase-2			−1.99	1.51
Fructose-1,6-bisphosphatase isozyme 2		1.62		
Glucose-6-phosphate isomerase-1				3.10
**Protein synthesis/catabolism/modification**				
40S ribosomal protein S9-3		−1.51		1.77
60S ribosomal protein L13a-acute pahse protein				2.14
60S ribosomal protein L32-2		−1.88		2.08
Cathepsin D				1.66
Proteasome activator complex subunit 2	1.60		1.83	
Proteasome subunit alpha type 7-2				−3.84
Proteasome subunit beta type 9 precursor	2.01	1.49		1.88
Serine protease-like protein-1		2.23		1.78
Ubiquitin		−3.73		−265.03
**Muscle development/cytoskeleton**				
Actin, alpha skeletal 3	1.73	−3.53		1.87
Creatine kinase, M-3		−2.75		
Myosin heavy chain, skeletal, adult 1-2		−3.32		−1.51
Myosin light chain 2-2	1.77	−5.94		3.20
Parvalbumin alpha-3	1.82	−74.54	1.99	2.38
Troponin I-4, fast skeletal muscle	1.72	−5.54	1.58	3.76
Tropomyosin alpha 3 chain-2		−2.11		2.55
**Immune response**				
Beta-2-microglobulin-1	2.13		1.52	1.56
CC chemokine SCYA110-2	−2.14		−2.97	
Ferritin heavy chain-1				1.62
Ig kappa chain V-IV region B17-2	1.58	3.41		
Lysozyme C precursor		1.59		2.17
Macrophage receptor MARCO	1.60			−1.59
MHC class 1b antigen	2.45		1.64	2.36
MHC class II invariant chain-like protein 1	1.60	2.19		2.06
**Lipid metabolism**				
Apolipoprotein A-I-1				4.56
Apolipoprotein E-2		1.72		
Fatty acid-binding protein-1	−1.62	1.68	−1.64	−2.17
Acyl-Coenzyme A dehydrogenase, long chain		2.46		
**Mitochondrion/electron transport**				
ATP synthase coupling factor 6, mitochondrial		2.19		
Cytochrome b-1		1.73		−1.71
Cytochrome oxidase subunit III-2		2.89		
NADH dehydrogenase subunit 5-1		7.21	1.53	1.89

^a^ Data shown represent mean fold change (FC) over control. Significantly up- and down-regulated genes (*p* < 0.01, Student’s *t*-test, 12 spot replicates per gene, −1.5 < FC > 1.5) are highlighted with a color scale.
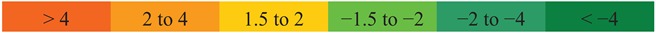

LPS administration in trout also caused significant changes in the mRNA expression levels of genes involved in protein metabolism, as shown by the large number of DEGs involved in protein biosynthesis as well as in protein modification and catabolism. In particular, this functional category included DEGs coding for several ribosomal proteins (e.g., 40S, 60S) in both types of muscle. In fast muscle, most of these genes were down-regulated at 24 h and up-regulated at 72 h, contrary to the situation in slow muscle (Table 2, Tables S4 and S5). Among genes involved in protein modification and catabolism, we highlight the altered expression of cathepsin D, proteasome subunits, serine proteases and ubiquitin in fast and slow muscle in response to LPS administration. Interestingly, LPS administration also caused changes in the functional category of muscle development in trout fast and slow muscle (Figure 2 and Figure 3) that included genes coding for proteins participating in the contractile apparatus of skeletal muscle fibers (e.g., alpha actin, myosin heavy chain 1, myosin light chain 2, troponin I-4, tropomyosin alpha 3) and in the regulation of contraction (e.g., parvalbumin alpha-3, creatine kinase M-3) (Table 2, Tables S2 to S5). Specifically, most DEGs involved in muscle development were up-regulated in fast muscle of LPS-injected trout at 24 and 72 h, whereas they were up-regulated in slow muscle at 24 h but strongly down-regulated at 72 h. The parallel increase in the expression of genes involved in protein synthesis and muscle development in fast muscle in response to LPS suggests that endotoxin administration in fish may induce metabolic and structural changes that may be linked to a muscle remodeling process. However, we cannot discard the possibility that the rate of protein synthesis may not vary concurrently with gene expression in skeletal muscle of LPS-injected trout, as it has been suggested for the rat skeletal muscle during sepsis [35]. Nevertheless, the notion that LPS may induce muscle remodeling is reinforced by data on the increased expression of genes involved in protein degradation in fast and slow muscle of LPS-injected trout. Similar results, although with some differences in time-related expression changes, were reported in a previous study from our group on the muscle transcriptomic response of another teleost species, the seabream (*Sparus aurata*), to LPS administration [18]. Therefore, we hypothesize that an acute administration of LPS in fish may result in the preservation of fast muscle that may otherwise be possibly used as fuel [36]. However, the different response of slow muscle to LPS administration in trout (*i.e.*, decreased expression of genes involved in protein synthesis and in muscle development) is reminiscent of an atrophy phenotype, as previously described in this species [37]. Given the observed alteration in the expression of genes important for skeletal muscle function, it would be important to determine in future studies if an acute challenge with LPS can affect swimming performance in trout and/or muscle contractile characteristics. In contrast to the acute effects of LPS described here, a chronic stimulation with LPS in trout (over 43 days) was reported to reduce skeletal muscle growth, although without changes in the mRNA levels of growth marker genes (e.g., myosins) [38]. Therefore, the results from these studies suggest that inflammatory conditions in fish could result in alterations in protein metabolism that could eventually affect muscle growth.

Interestingly, an acute LPS administration in trout, previously shown to induce a potent inflammatory response in both the head kidney and in the spleen [11,27] involving increased expression of the antigen presentation machinery and a vast array of anti-bacterial products, resulted in changes in the expression of genes involved in the immune response in fast and slow muscle. The effects of LPS were particularly noticeable at 72 h after LPS administration in both types of muscle, causing an up-regulation of the expression of genes involved in antigen presentation (e.g., MCH class I and II, beta-2-microglobulin-1), in the degradation of mucopolysaccharide from the bacterial wall (e.g., lysozyme C) and in iron sequestering (e.g., ferritin heavy chain-1). The general up-regulation of immune-related genes observed in this study is consistent with the previously reported increased expression of immune genes in trout skeletal muscle after chronic LPS treatment, including lysozyme [38]. However, it is not known if the detected immune genes are expressed in skeletal muscle cells or in immune cells present in this tissue. In mammals, the expression of immune molecules by macrophages infiltrated in skeletal muscle appears to be essential during tissue repair, stimulating myogenesis and fiber growth [39]. These types of mechanisms cannot be discarded in trout, although such episodes appear to be significant, at least in mammals, only in lesioned muscle [40,41] and probably do not reflect the changes in the gene expression profile of skeletal muscle identified in our study. In this respect, several studies have shown that the response of the mammalian skeletal muscle to endotoxin exposure stimulates antigen presentation, which elicits an immune response by inducing the production of immune molecules such as MHC molecules, adhesion molecules and cytokines [4]. In mammals it is well known that the skeletal muscle possesses many of the components of the innate immune system, including the presence of toll-like receptors and the ability to produce cytokines [42]. In the mammalian skeletal muscle, binding of LPS to its receptor (TLR-4) promotes the synthesis and secretion of inflammatory cytokines such as TNFα [43], which has multiple metabolic effects, including the regulation of lipid metabolism through the activation of lipolysis [44,45,46]. Despite the lack of TLR4 in the rainbow trout and the unresolved question of whether LPS action is mediated by TLRs in fish [47], a conservation of this inflammatory response pathway may be present in trout tissues because LPS directly increases TNFα mRNA levels and TNFα protein secretion by cultured trout macrophages [13,48] and *in vivo* administration of LPS increases TNFα mRNA levels in trout skeletal muscle and ovarian tissue [33,38]. Interestingly, LPS administration *in vivo* in trout is known to increase the basal lipolytic rate in isolated trout adipocytes *in vivo* and TNFα directly stimulates lipolysis in trout adipocytes *in vitro* [49]. In the present study, the observed decrease in the plasma levels of triglycerides in response to LPS administration in trout (at 24 h) is consistent with the notion of a catabolic role of LPS on lipid metabolism that may be mediated by TNFα. Although we do not know whether LPS can act directly on skeletal muscle cells, we hypothesize that LPS administration in trout may have stimulated TNFα production that, in turn, may have increased the entry of lipids into skeletal muscle, primarily in slow muscle as suggested by the increased expression of genes involved in lipid transport (e.g., apolipoproteins A-I and E-2, fatty acid binding protein-1), and their catabolism, as suggested by the increased expression of Acyl-CoA dehydrogenase (Table 2), an enzyme that catalyzes the first step of mitochondrial fatty acid beta-oxidation. Our results suggest that lipids could have a preponderant role fulfilling the increase in energy demand during the early stages in the response of trout to an acute endotoxin exposure. Additionally, triglycerides included in circulating lipoproteins may have an important role against LPS-induced toxicity and have been considered an important component of an innate, non-adaptive immune response of the host to a bacterial infection [50].

#### 3.2.1. Muscle Type-Specific Responses to LPS Administration

**Figure 4:**
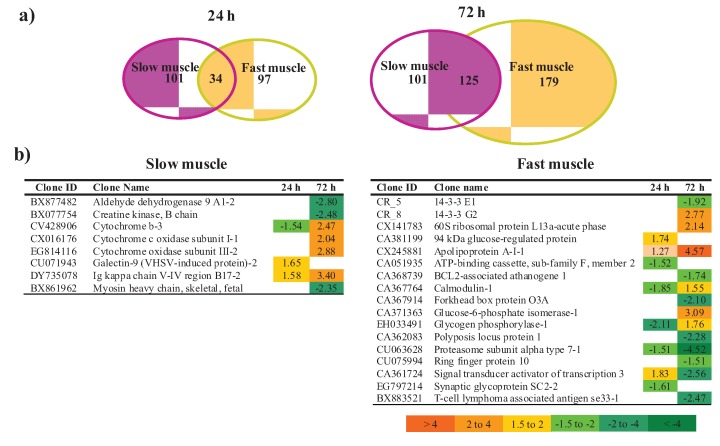
Muscle type-specific responses to lipopolysaccharide (LPS) administration in trout. (**a**) Venn diagrams showing the number of slow skeletal muscle differentially expressed genes (DEGs; *p* < 0.01), fast skeletal muscle DEGs, and common DEGs at 24 and 72 h after LPS administration in trout; (**b**) List of selected DEGs specific for slow and fast muscle in response to LPS administration. Data shown represent mean fold change (FC) over control. Significantly up- and down-regulated genes (*p* < 0.01, Student’s *t*-test, 12 spot replicates per gene, −1.5 < FC > 1.5) are highlighted with a color scale.

In order to identify specific responses to LPS in each of the two types of skeletal muscle, we performed a comparative analysis at 24 and 72 h after LPS administration (Figure 4a). At 24 h after LPS administration, 101, 97 and 34 DEGs were found only in slow muscle, only in fast muscle and in both tissues, respectively. At 72 h, 101, 125 and 179 DEGs were found only in slow muscle, only in fast muscle and in both tissues, respectively. LPS administration produces in trout a notable contrasting effect in the expression of genes related to protein synthesis (e.g., several ribosomal components) between both muscle types, particularly after 72 h, that were down-regulated in slow skeletal muscle and up-regulated in fast skeletal muscle. Furthermore, such changes were accompanied by differential expression of several genes involved in protein modification and catabolism, that included cathepsins, proteasome subunits, proteases and ubiquitin related proteins in both types of muscle. We note the specific expression in fast muscle of genes involved in protein synthesis (e.g., ribosomal proteins), calcium storage (e.g., calmodulin-1), carbohydrate metabolism (e.g., glycogen phosphorylase-1 and glucose phosphate isomerase-1) and lipid metabolism (e.g., apolipoprotein A), all significantly up-regulated at 72 h after LPS administration. Moreover, forkhead box protein O3A, an important factor in energy balance, was also expressed exclusively in fast muscle and was down-regulated (Figure 4b). Among genes that were differentially expressed exclusively in red muscle we highlight creatine kinase B and several forms of cytochromes involved in energy generation (e.g., cytochrome b-3, cytochrome c oxidase subunits I-1 and III-2), together with skeletal myosin heavy chain involved in contractile activity in muscle (Figure 4b). Parallel to this response, the expression of genes encoding contractile and structural elements in skeletal muscle (troponin, tropomyosin, actin, and myosin) was down-regulated in slow skeletal muscle but up-regulated in fast skeletal muscle, reinforcing the idea that LPS administration exerts a muscle-specific transcriptomic response. In addition to evaluating the tissue-specific responses to LPS as described above, in order to understand how fast and slow skeletal muscles respond to LPS it is also useful to examine the expression of those genes that appear differentially expressed in both tissues, regardless of the direction of change (Tables S5 and S6). Most notably, this type of analysis, particularly at 72 h after LPS administration, clearly evidences the contrasting regulation of structural and contractile elements between the two types of muscle, with up-regulation in fast muscle and down-regulation in slow muscle (Table S6).

### 3.3. Proteomic Analysis of Fast Skeletal Muscle in Response to LPS Administration in Rainbow Trout

**Figure 5:**
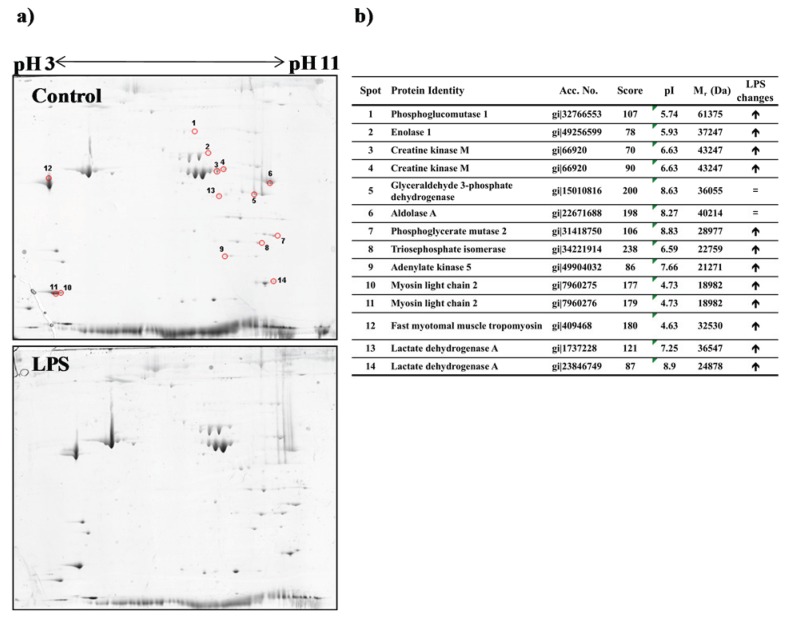
Proteomic changes in trout fast skeletal muscle after lipopolysaccharide (LPS) administration. (**a**) Representative bi-dimensional Coomassie stained 2-DE gels of pooled fast skeletal muscle samples from trout injected with saline (Control; *n* = 3) or LPS (*n* = 3), performed in triplicate. In the control gel, the spots circled in red indicate proteins identified by MALDI/TOF; (**b**) Identification of proteins separated by 2-DE. Database accession numbers are indicated as a GI number. MASCOT score greater than 72 indicates that the protein identification is significant (*p* < 0.05). The qualitative direction of change of identified proteins in LPS *versus* control gels is indicated by arrows. = indicates absence of visual changes in spot intensity.

In order to integrate the described transcriptomic response of skeletal muscle to LPS administration with possible changes at the protein level, we performed a proteomic analysis of the trout fast muscle at 24 h after the LPS challenge, given that fast muscle represents most of the consumable flesh in fish. Using two-dimensional electrophoresis (2-DE) we detected ~100 protein spots in the fast muscle of trout, with M_r_ values between 20,000–120,000 and pI values between 4–9 (Figure 5a). Comparison between LPS and control samples among three replicates showed high similarity in the spots analyzed. Analysis of gels from these experiments showed an induction of several proteins in fast muscle in response to LPS challenge (*p* < 0.005), as shown by the changes in the intensity in most of the spots visualized with Coomassie staining (Figure 5a).

Fourteen protein spots obtained from Coomassie-stained gels were excised and analyzed by MALDI-TOF-MS for identification. The selected spots showed qualitative differences in intensity or pI between control and LPS-treated trout and corresponded to proteins engaged in muscle contraction (e.g., myosin light chain and fast myotomal muscle tropomyosin), enzymes involved in energy metabolism, mainly glycolysis (e.g., phosphoglucomutase, enolase, glyceraldehyde 3-phosphate dehydrogenase, aldolase, phosphoglycerate mutase, triosephosphate isomerase and lactate dehydrogenase), and energy homeostasis (e.g., creatine kinase and adenylate kinase) (Figure 5b). In some cases (e.g., lactate dehydrogenase, creatine kinase and enolase), the analysis of the protein spots reflected changes in the mobility of these proteins as a consequence of differences in pI or M_r_. The exception to these described changes in proteins from muscle samples from LPS-treated trout occurred with the enzymes glyceraldehyde 3-phosphate dehydrogenase and aldolase, whose spots remained unchanged in pI, M_r_ or intensity when compared to control trout (Figure 5b). Interestingly, the increase in the protein content of several glycolytic enzymes (phosphoglucomutase, enolase, phosphoglycerate mutase, and triosephosphate isomerase) in fast muscle of trout in response to LPS administration (at 24 h after injection) is related to the observed up-regulation, as assessed by microarray analysis, of the expression of genes involved in glycogen breakdown (glycogen phosphorylases) and glycolysis (enolase and glyceraldehyde-3-phosphate dehydrogenase). This is also supported by the decrease in glycogen levels in fast skeletal muscle in LPS-injected fish at 72 h, suggesting an increase in the utilization of fast muscle glycogen stores. Although plasma levels of glucose were not significantly altered in response to LPS administration, it is possible that glucose utilization for energy derivation was increased in fast muscle, as suggested by the significantly altered expression of genes involved in carbohydrate metabolism, nutrient oxidation and ATP synthesis-coupled electron transport in this tissue (Figure 3 and Figure S2). We hypothesize that the observed increase in glycogenolysis in fast muscle of trout may be caused by the higher glucose demands imposed after endotoxin exposure, as has been suggested in the mammalian muscle [51]. However, it can be argued that the transcriptomic and proteomic analyses of trout fast muscle at 24 h after LPS exposure suggest a diverging pattern in glycolysis, as shown by the down-regulation at the gene expression level and the increase in protein content of enzymes in this metabolic pathway. It is well known that changes in gene expression and protein levels may not necessarily correlate, sustaining the necessity to complement transcriptomic with proteomic approaches [52,53]. However, the decrease in the mRNA expression levels of genes coding for glycolytic enzymes was reverted at 72 h after LPS exposure, reinforcing the importance of this metabolic pathway in providing energy to the trout fast muscle. Similar contrasting conclusions between transcriptomic and proteomic studies were obtained in zebrafish subjected to hypoxia [54,55] as the gene expression program may display a delay with respect to protein synthesis. Overall, the changes in metabolic enzymes at the protein and mRNA level in fast muscle in response to LPS administration are suggestive of an enhanced importance for carbohydrate utilization in fast muscle, the largest tissue in the body, under proinflammatory conditions such as those elicited by LPS administration. In addition to glycolysis, contractile elements and regulatory factors in fast muscle also showed parallel transcriptomic and proteomic changes. In particular, myosin light chain 2, a protein identified in two different spots as more abundant under LPS treatment, also showed increased mRNA expression levels (myosin light chain 2.1 and 2.2) by microarray analysis in fast muscle at 72 h after LPS administration (Table S5). These changes in the expression of specific contractile elements in fast muscle both at the proteomic and transcriptomic levels are in agreement with the general increase of the expression of genes involved in functional categories related to muscle contraction and development in fast skeletal muscle in response to LPS administration in rainbow trout. A similar up-regulation of functional categories related to contractile and structural elements of fast skeletal muscle was observed in the gilthead seabream in response to LPS administration [18] and points towards a common response of the fish fast muscle to a challenge with LPS possibly to safeguard proteins involved in muscle contraction and, therefore, important for swimming performance.

## 4. Conclusions

In the present study, by applying a combination of transcriptomic and proteomic analyses to investigate the regulation of skeletal muscle function in rainbow trout subjected to a proinflammatory insult, we show that an acute treatment with LPS in rainbow trout results in alterations in metabolic parameters in the blood and in transcriptomic and proteomic changes in skeletal muscle. Most notably, our results strongly suggest that LPS administration may have promoted carbohydrate metabolism in fast muscle to generate energy from glucose by mobilizing glycogen stores and by stimulating glycolysis in fast muscle. Similarly, our results suggest that LPS may have caused an increase in the utilization of lipids as a source of energy. In view of these apparent catabolic effects, it is surprising that LPS administration induced the expression of genes involved in protein synthesis and degradation coupled with increased expression of sarcomeric contractile elements in fast muscle, perhaps as part of a compensatory muscle remodeling process in the face of a metabolic challenge. Overall, the results from this study indicate that LPS administration in rainbow trout has a significant impact on skeletal muscle physiology and may alter whole body energy homeostasis. Further studies are needed to understand how immune challenges can affect energy use and growth in fish.

## Supplementary Files

Supplementary File 1

## References

[1] Degens H. (2010). The role of systemic inflammation in age-related muscle weakness and wasting. Scand. J. Med. Sci. Sports.

[2] Rectenwald J.E., Moldawer L.L., Preedy V.R., Peters T.J. (2002). Skeletal muscle and cytokines in sepsis and severe injury. Skeletal Muscle: Pathology, Diagnosis and Management of Disease.

[3] Frost R.A., Lang C.H. (2008). Regulation of muscle growth by pathogen associated molecules. J. Anim. Sci..

[4] Wiendl H., Hohlfeld R., Kieseier B. (2005). Immunobiology of muscle: Advances in understanding an immunological microenvironment. Trends Immunol..

[5] Nagaraju K. (2001). Immunological capabilities of skeletal muscle cells. Acta Physiol. Scand..

[6] Read R.C., Wyllie D.H. (2001). Toll receptors and sepsis. Curr. Opin. Crit. Care.

[7] Michie H.R. (1996). Metabolism of sepsis and multiple organ failure. World J. Surg..

[8] Iliev D.B., Roach J.C., MacKenzie S., Planas J.V., Goetz F.W. (2005). Endotoxin recognition: In fish or not in fish?. FEBS Lett..

[9] Palti Y. (2011). Toll-like receptors in bony fish: From genomics to function. Dev. Comp. Immunol..

[10] MacKenzie S., Iliev D., Liarte C., Koskinen H., Planas J.V., Goetz F.W., Mölsä H., Krasnov A., Tort L. (2006). Transcriptional analysis of LPS-stimulated activation of trout (*Oncorhynchus mykiss*) monocyte/macrophage cells in primary culture treated with cortisol. Mol. Immunol..

[11] MacKenzie S., Balasch J.C., Novoa B., Ribas L., Roher N., Krasnov A., Figueras A. (2008). Comparative analysis of the acute response of the trout, *O. mykiss*, head kidney to *in vivo* challenge with virulent and attenuated infectious hematopoietic necrosis virus and LPS-induced inflammation. BMC Genomics.

[12] Swain P., Nayak S.K., Nanda P.K., Dash S. (2008). Biological effects of bacterial lipopolysaccharide (endotoxin) in fish: A review. Fish Shellfish Immunol..

[13] MacKenzie S., Planas J.V., Goetz F.W. (2003). LPS-Stimulated expression of a tumor necrosis factor-a mRNA in primary trout monocytes and *in vitro* differentiated macrophages. Dev. Comp. Immunol..

[14] Iliev D.B., Liarte C.Q., MacKenzie S., Goetz F.W. (2005). Activation of rainbow trout (*Oncorhynchus mykiss*) mononuclear phagocytes by different pathogen associated molecular pattern (PAMP) bearing agents. Mol. Immunol..

[15] Moyes C.D., West T.G., Hochachka P.W., Mommsen T.P., Hochachka P.W., Mommsen T.P. (1995). Exercise metabolism of fish. Metabolic Biochemistry.

[16] Bone Q., Hoar W.S., Randall D.J. (1978). Locomotor muscle. Fish Physiology.

[17] Altringham J.D., Ellerby D.J. (1999). Fish swimming: patterns in muscle function. J. Exp. Biol..

[18] Kaitetzidou E., Crespo D., Vraskou Y., Antonopoulou E., Planas J.V. (2012). Transcriptomic response of skeletal muscle to lipopolysaccharide in the gilthead seabream (*Sparus aurata*). Mar. Biotechnol..

[19] Magnoni L.J., Crespo D., Ibarz A., Blasco J., Fernández-Borràs J., Planas J.V. (2013). Effects of sustained swimming on the red and white muscle transcriptome of rainbow trout (*Oncorhynchus mykiss*) fed a carbohydrate-rich diet. Comp. Biochem. Physiol. A Mol. Integr. Physiol..

[20] Hassid W.Z., Abraham S., Colowick S.P., Kaplan N.O. (1957). Chemical procedures for the analysis of polysaccharides. Methods in Enzymology.

[21] Koskinen H., Pehkonen P., Vehniainen E., Krasnov A., Rexroad C., Afanasyev S., Molsa H., Oikari A. (2004). Response of rainbow trout transcriptome to model chemical contaminants. Biochem. Biophys. Res. Commun..

[22] Krasnov A., Koskinen H., Pehkonen P., Rexroad C., Afanasyev S., Molsa H. (2005). Gene expression in the brain and kidney of rainbow trout in response to handling stress. BMC Genomics.

[23] MacKenzie S., Montserrat N., Mas M., Acerete L., Tort L., Krasnov A., Goetz F.W., Planas J.V. (2006). Bacterial lipopolysaccharide induces apoptosis in the trout ovary. Reprod. Biol. Endocrinol..

[24] Jorgensen S.M., Hetland D.L., Press C.M., Grimholt U., Gjøen T. (2007). Effect of early infectious salmon anaemia virus (ISAV) infection on expression of MHC pathway genes and type I and II interferon in Atlantic salmon (*Salmo salar* L.) tissues. Fish Shellfish Immunol..

[25] Schiotz B.L., Jorgensen S.M., Rexroad C., Gjoen T., Krasnov A. (2008). Transcriptomic analysis of responses to infectious salmon anemia virus infection in macrophage-like cells. Virus Res..

[26] Skugor S., Glover K.A., Nilsen F., Krasnov A. (2008). Local and systemic gene expression responses of Atlantic salmon (*Salmo salar* L.) to infection with the salmon louse (*Lepeophtheirus salmonis*). BMC Genomics.

[27] Djordjevic B., Skugor S., Jorgensen S.M., Overland M., Mydland L.T., Krasnov A. (2009). Modulation of splenic immune responses to bacterial lipopolysaccharide in rainbow trout (*Oncorhynchus mykiss*) fed lentinan, a beta-glucan from mushroom *Lentinula edodes*. Fish Shellfish Immunol..

[28] Ashburner M., Ball C.A., Blake J.A., Botstein D., Butler H., Cherry J.M., Davis A.P., Dolinski K., Dwight S.S., Eppig J.T. (2000). Gene ontology: Tool for the unification of biology. The Gene Ontology Consortium. Nat. Genet..

[29] Livak K.J., Schmittgen T.D. (2001). Analysis of relative gene expression data using real-time quantitative PCR and the 2-[delta][delta]CT method. Methods.

[30] Goetz F.W., Iliev D.B., McCauley L.A.R., Liarte C.Q., Tort L.B., Planas J.V., MacKenzie S. (2004). Analysis of genes isolated from lipopolysaccharide-stimulated rainbow trout (*Oncorhynchus mykiss*) macrophages. Mol. Immunol..

[31] Goldstein S.A., Elwyn D.H. (1989). The effects of injury and sepsis on fuel utilization. Annu. Rev. Nutr..

[32] Marik P.E., Raghavan M. (2004). Stress-hyperglycemia, insulin and immunomodulation in sepsis. Intens. Care Med..

[33] Crespo D., Bonnet E., Roher N., Mackenzie S.A., Krasnov A., Goetz F.W., Bobe J., Planas J.V. (2010). Cellular and molecular evidence for a role of tumor necrosis factor alpha in the ovulatory mechanism of trout. Reprod. Biol. Endocrinol..

[34] Callahan L.A., Supinski G.S. (2004). Downregulation of diaphragm electron electron transport chain and glycolitic enzyme expression in sepsis. J. Appl. Physiol..

[35] Macallan D.C., Cook E.B., Preedy V.R., Griffin G.E. (1996). The effect of endotoxin on skeletal muscle protein gene expression in the rat. J. Biochem. Cell Biol..

[36] Van den Thillart G. (1986). Energy metabolism of swimming trout (*Salmo gairdneri*). J. Comp. Physiol. B.

[37] Salem M., Kenney P.B., Rexroad C.E., Yao J. (2006). Microarray gene expression analysis in atrophying rainbow trout muscle: A unique nonmammalian muscle degradation model. Physiol. Genom..

[38] Johansen K.A., Sealey W.M., Overturf K. (2006). The effects of chronic immune stimulation on muscle growth in rainbow trout. Comp. Biochem. Physiol. B.

[39] Arnold L., Henry A., Poron F., Baba-Amer Y., van Rooijen N., Plonquet A., Gherardi R.K., Chazaud B. (2007). Inflammatory monocytes recruited after skeletal muscle injury switch into antiinflammatory macrophages to support myogenesis. J. Exp. Med..

[40] McLennan I.S. (1996). Degenerating and regenerating skeletal muscles contain several subpopulations of macrophages with distinct spatial and temporal distributions. J. Anat..

[41] Pimorady-Esfahani A., Grounds M.D., McMenamin P.G. (1997). Macrophages and dendritic cells in normal and regenerating murine skeletal muscle. Muscle Nerve.

[42] Lang C.H., Silvis C., Deshpande N., Nystrom G., Frost R.A. (2003). Endotoxin stimulates *in vivo* expression of inflammatory cytokines tumor necrosis factor alpha, interleukin-1[beta], -6, and high-mobility-group protein-1 in skeletal muscle. Shock.

[43] Frost R.A., Nystrom G.J., Lang C.H. (2002). Lipopolysaccharide regulates proinflammatory cytokine expression in mouse myoblasts and skeletal muscle. Am. J. Physiol. Regul. Integr. Comp. Physiol..

[44] Grunfeld C., Feingold K.R. (1992). Tumor necrosis factor, interleukin, and interferon induced changes in lipid metabolism as part of host defense. Proc. Soc. Exp. Biol. Med..

[45] Tracey K.J., Cerami A. (1993). Tumor necrosis factor, other cytokines and disease. Annu. Rev. Cell Biol..

[46] Hardardóttir I., Grunfeld C., Feingold K.R. (1994). Effects of endotoxin and cytokines on lipid metabolism. Curr. Opin. Lipidol..

[47] Zhang J., Kong X., Zhou C., Li L., Nie G., Li X. (2014). Toll-Like receptor recognition of bacteria in fish: Ligand specificity and signal pathways. Fish Shellfish Immunol..

[48] Roher N., Callol A., Planas J.V., Goetz F.W., Mackenzie S.A. (2011). Endotoxin recognition in fish results in inflammatory cytokine secretion not gene expression. Innate Immun..

[49] Albalat A., Liarte C., MacKenzie S., Tort L., Planas J.V., Navarro I. (2005). Control of adipose tissue lipid metabolism by tumor necrosis factor alpha in rainbow trout (*Oncorhynchus mykiss*). J. Endocrinol..

[50] Barcia A.M., Harris H.W. (2005). Triglyceride-rich lipoproteins as agents of innate immunity. Clin. Infect. Dis..

[51] Tessier J.-P., Thurner B., Jungling E., Luckhoff A., Fischer Y. (2003). Impairment of glucose metabolism in hearts from rats treated with endotoxin. Cardiovasc. Res..

[52] Celis J.E., Kruhøffer M., Gromova I., Frederiksen C., Ostergaard M., Thykjaer T., Gromov P., Yu J., Pálsdóttir H., Magnusson N. (2000). Gene expression profiling: Monitoring transcription and translation products using DNA microarrays and proteomics. FEBS Lett..

[53] Forné I., Castellana B., Marín-Juez R., Cerdà J., Abián J., Planas J.V. (2011). Transcriptional and proteomic profiling of flatfish (*Solea senegalensis*) spermatogenesis. Proteomics.

[54] Bosworth C., Chou C.W., Cole R.B., Rees B.B. (2005). Protein expression patterns in zebrafish skeletal muscle: Initial characterization and the effects of hypoxic exposure. Proteomics.

[55] Ton C., Stamatiou D., Liew C.-C. (2003). Gene expression profile of zebrafish exposed to hypoxia during development. Physiol. Genomics.

