# Supplementary material for: In Vivo Molecular Responses of Fast and Slow Muscle Fibers to Lipopolysaccharide in a Teleost Fish, the Rainbow Trout (Oncorhynchus mykiss)
Source: Biology (Basel). 2015 Feb 4;4(1):67–87. doi: 10.3390/biology4010067 (PMC4381218; doi:10.3390/biology4010067)
Supplement: Supplementary File 1 [file biology-04-00067-s001.pdf]

Supplementary Material

a)

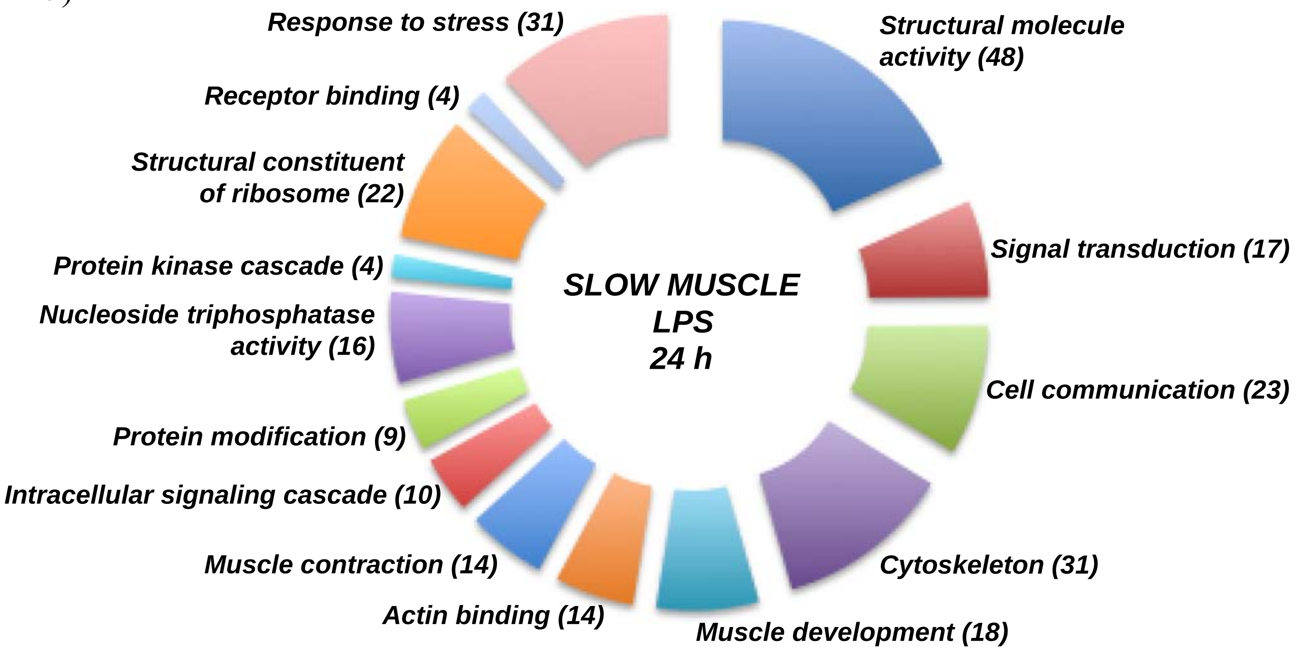

b)

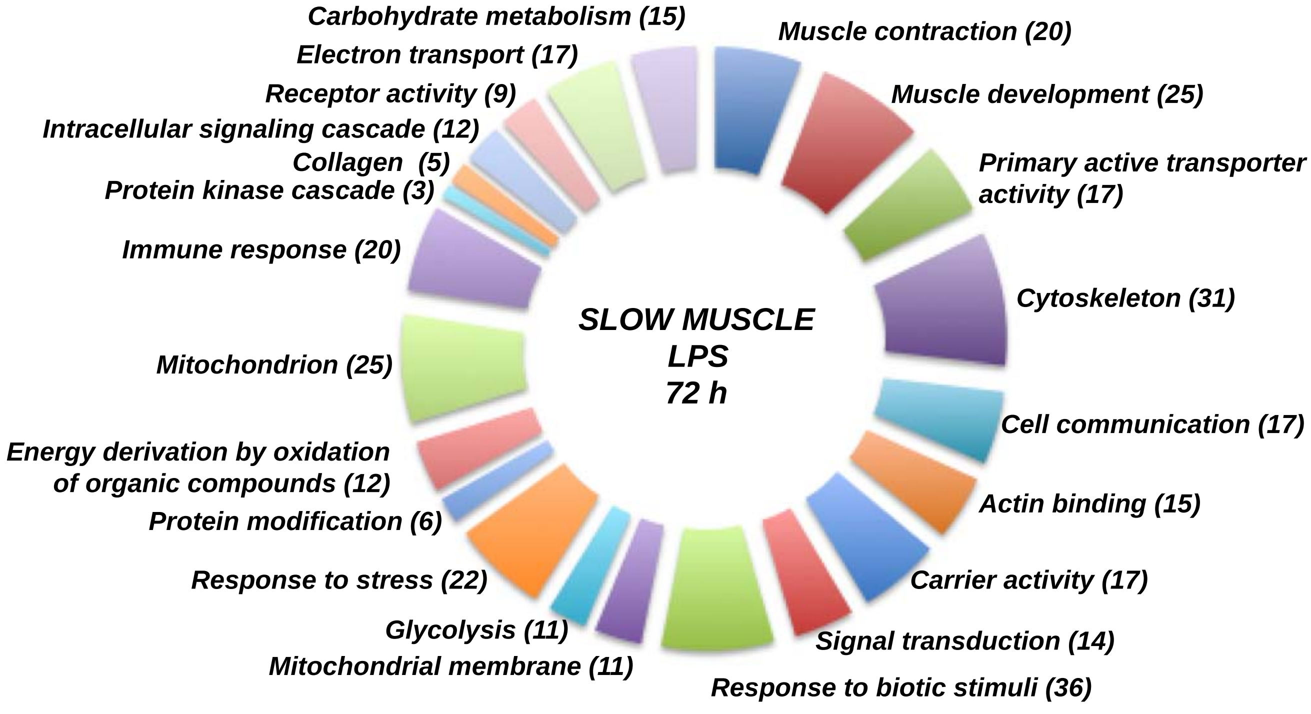

Figure S1. Over represented gene ontology functional classes in slow skeletal muscle in trout at 24 h (**a**) and 72 h (**b**) after LPS administration. Functional categories with Yates corrected Chi squared ($p < 0.05$) were selected. The number of regulated genes for each category is shown in parenthesis.

a)

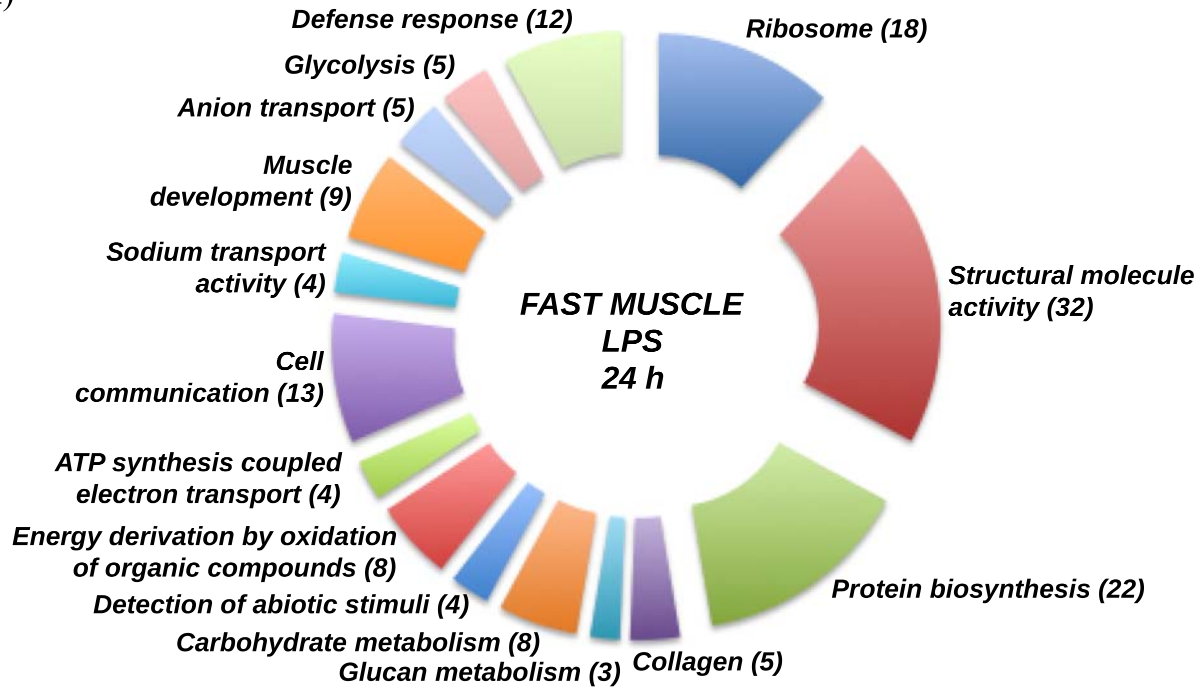

b)

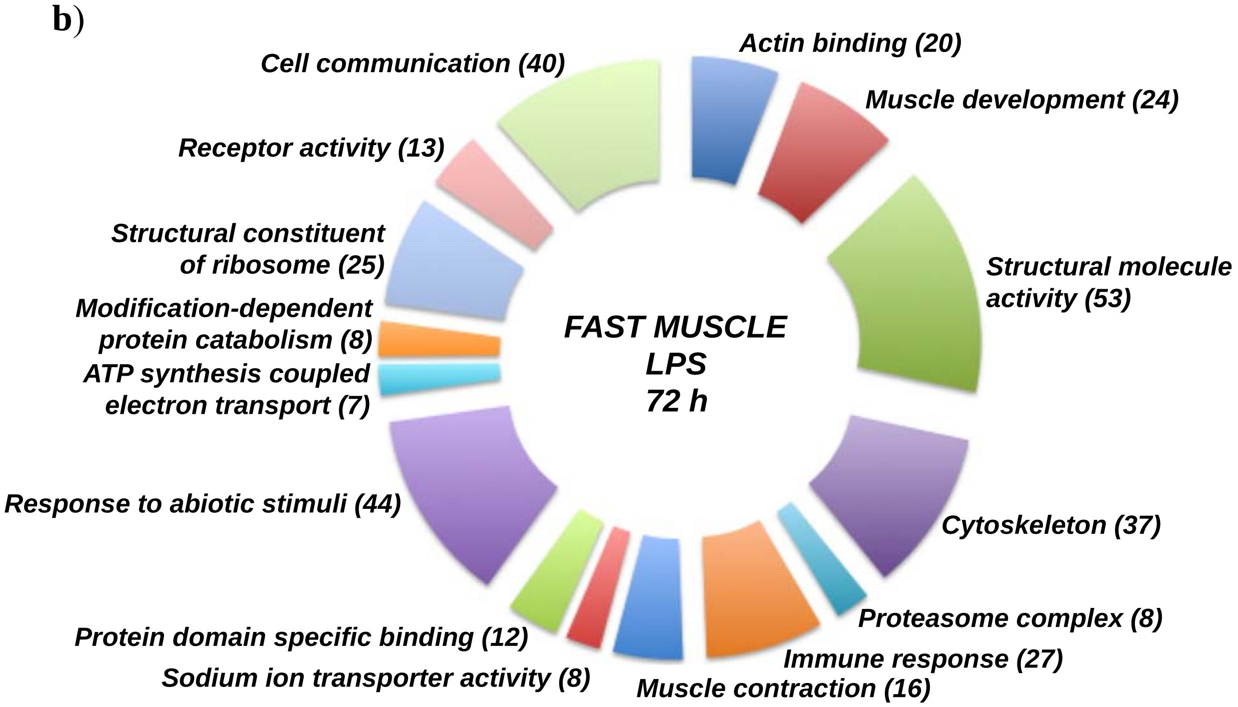

Figure S2. Over represented gene ontology functional classes in fast skeletal muscle in trout at 24 h (**a**) and 72 h (**b**) after LPS administration. Functional categories with Yates corrected Chi squared ($p < 0.05$) were selected. The number of regulated genes for each category is shown in parenthesis.

Table S1. Sequences of primers used in gene expression analyses by qPCR.

GenBank ID	Clone Name	Primer Sequence (5'-3')
CB511095	Glyceraldehyde-3-phosphate dehydrogenase 3	(F) GGTTGGTTGCGTTACTCCTTGGT<br>(R) CCAGTTACGACGCCATCAAGAAG
BX074486	Heat shock protein HSP 90-beta-2	(F) CACAATGGGCTACATGATGG<br>(R) CCTGTAGATGCGGTTGGAGT
CX146261	Myosin light chain 1, skeletal muscle isoform	(F) CAGGGGGAAAATGGACTATGAGAT<br>(R) CAGGGGACAAGACAACAAGAGC
CU069718	Serine protease-like protein-1	(F) ACCAAAAACATGCTGTGTGC<br>(R) CCCTCCATTTGAAGTGATCC
DY467707	Tropomyosin alpha 3 chain-2	(F) CTGAGAAGGCGGCTGATGAGT<br>(R) CCTCCTCGTATTTGCGGTCG
CA039449	Troponin I-4, fast skeletal muscle	(F) GGGTGACGAAGGCAGACAAGGA<br>(R) CATCCACTCCCTCCTCTTTGACTT
ES325822	Parvalbumin 3	(F) GCAAGTCCAGCGATGATGTGAAGA<br>(R) GCCTTGGTCTCAGCGTCAGTCA
CA371001	Very-long-chain acyl-CoA synthetase	(F) GCCAGCAGACAGAGAAGAAGAGAC<br>(R) TCCACCTGAATGTATCTCCGACTC
AF308735	18S ribosomal RNA	(F) CGGAGGTTCGAAGACGATCA<br>(R) TCGCTAGTTGGCATCGTTTAT

(F) forward; (R) reverse.

Table S2. Differentially expressed genes in slow skeletal muscle at 24 h after lipopolysaccharide (LPS) administration in rainbow trout.

Clone ID	Clone Name	FC	*p*-Value
est02f04	Tubulin alpha-3 chain	2.74	0.00000
est04e04	MHC class 1b antigen	2.45	0.00000
utu01e06	Troponin I-2, fast skeletal muscle	2.43	0.00000
HKT0001_B03	Alpha 2 type I collagen-1	2.16	0.00000
EXOB4_A11	Tubulin alpha-ubiquitous chain	2.16	0.00001
ENH2_F09	Beta-2-microglobulin-1	2.13	0.00000
utu02b11	Collagen a3(I)-2	2.11	0.00000
utu04c11	Collagen alpha 1(I) chain-2	2.06	0.00000
est02f12	Proteasome subunit beta type 9 precursor	2.01	0.00003
HKT0001_H03	Microtubule-associated protein RP/EB	2.01	0.00002
utu03e06	Parvalbumin alpha-3	1.81	0.00000
utu02f09	Myosin light chain 2-2	1.77	0.00000
est02b02	PEST-containing nuclear protein	1.74	0.00000
utu01g11	Actin, alpha skeletal 3	1.73	0.00006
Hete0002_B08	Eukaryotic translation elongation factor 1 alpha 3	1.73	0.00251
HK0003_E07	Myosin light chain 2-1	1.73	0.00002
utu04a11	Troponin I-4, fast skeletal muscle	1.72	0.00001
CA368961	78 kDa glucose-regulated protein precursor	1.72	0.00063
utu03e10	Troponin I-3, fast skeletal muscle	1.66	0.00020

Table S2. *Cont.*

Clone ID	Clone Name	FC	*p*-Value
HK0001_A12	40S ribosomal protein S2	1.66	0.00001
EXOB4_G02	Galectin-9 (VHSV-induced protein)-2	1.65	0.00084
utu01e09	Embryonic alpha-type globin2+collagen alpha 2(1)	1.63	0.00000
est01g07	Proteasome activator complex subunit 2	1.60	0.00151
EXOB4_B09	Macrophage receptor MARCO	1.60	0.00141
EXOB2_A01	MHC class II invariant chain-like protein 1	1.60	0.00018
EXOB2_A11	Tubulin alpha-1 chain	1.60	0.00099
est02a11	Ig kappa chain V-IV region B17-2	1.58	0.00790
EXOB1_H01	Hypothetical-fish 42	1.57	0.00356
utu03f12	26S proteasome non-ATPase regulatory subunit 14	1.57	0.00026
CA378361	Ubiquitin ligase SIAH1	1.55	0.00193
utu01f04	Actin, alpha skeletal 2	1.55	0.00003
est03b12	Secretory granule proteoglycan core protein	1.52	0.00032
est04b04	Cytokeratin 8	1.51	0.00128
EXOB2_C01	Cofilin, muscle isoform	1.51	0.00066
est04f10	Hypothetical-fish 1	1.50	0.00030
est04e06	Caspase recruitment domain protein 4	1.50	0.00078
HST0001_D04	Alpha-globin I-2	−1.52	0.00521
HK0003_C07	Heterogeneous nuclear ribonucleoprotein A0	−1.53	0.00728
est04h11	Iodotyrosine dehalogenase protein	−1.53	0.00199
utu03h02	6-phosphofructokinase	−1.53	0.00043
HKT0001_H05	Cytochrome b-3	−1.54	0.00025
utu04b04	Hypothetical-fish 32	−1.54	0.00000
CA363480	Aquaporin-CHIP	−1.57	0.00076
EXOB3_H01	Matrix metalloproteinase-13	−1.58	0.00077
EXOB3_G08	Fatty acid-binding protein-1	−1.63	0.00020
EXOB2_B10	Hemoglobin beta chain	−1.65	0.00215
EXOB1_H06	CC chemokine SCYA110-2	−2.14	0.00000

> 4	2 to 4	1.5 to 2	−1.5 to −2	−2 to −4	< −4

A SFA 2.0 microarray platform was used for the gene expression analysis in slow skeletal muscle at 24 h after LPS exposure, according to the procedure described in Materials and Methods. Significantly up- and down-regulated genes ($p < 0.01$, Student's test, 12 spot replicates per gene, $-1.5 > FC < -1.5$) are highlighted with a color scale. Data shown represent mean fold change (FC).

Table S3. Differentially expressed genes in slow skeletal muscle at 72 h after lipopolysaccharide (LPS) administration in rainbow trout.

Clone ID	Clone Name	FC	*p*-Value
EXOB2_D06	NADH dehydrogenase subunit 5-1	7.22	0.00000
utu03f07	NADH dehydrogenase subunit 5-2	3.78	0.00000
utu01e09	Embryonic alpha-type globin2+collagen alpha 2(1)	3.70	0.00000
utu01h02	Cytochrome oxidase subunit III-3	3.63	0.00000
HKT0001_B03	Alpha 2 type I collagen-1	3.53	0.00000
EXOB1_H12	NADH dehydrogenase subunit 4	3.50	0.00000
est02a11	Ig kappa chain V-IV region B17-2	3.40	0.00000

Table S3. *Cont.*

Clone ID	Clone Name	FC	*p*-Value
P_46	ATPase 6	3.40	0.00000
est02f05	Cytochrome c oxidase subunit III-4	3.18	0.00000
EST1-3A_G02	NADH dehydrogenase subunit 2	3.06	0.00000
utu02a06	Collagen a3(I)-1	2.93	0.00000
utu01g04	Cytochrome oxidase subunit III-2	2.88	0.00000
utu04c11	Collagen alpha 1(I) chain-2	2.81	0.00000
HK0003_A03	Thymosin beta-4-2	2.60	0.00000
HKT0001_H05	Cytochrome b-3	2.47	0.00007
HK0001_H01	Acyl-Coenzyme A dehydrogenase, long chain,	2.47	0.00001
EXOB2_D09	GWSC6486	2.34	0.00000
est02f08	Serine protease-like protein-1	2.24	0.00000
EXOB2_A01	MHC class II invariant chain-like protein 1	2.19	0.00013
utu02e04	ATP synthase coupling factor 6, mitochondrial precursor	2.19	0.00000
CA348325	BAG-family molecular chaperone regulator-4	2.17	0.00000
est03e02	Hypothetical-fish 15	2.12	0.00002
HK0002_F06	Histone 3A-ATP synthase F0 6	2.10	0.00005
est02g11	Cytochrome c oxidase subunit I-1	2.04	0.00013
utu02b07	Cytochrome c oxidase subunit II	2.01	0.00001
utu03g04	Myosin heavy chain, cardiac muscle beta isoform	2.00	0.00000
CA382570	Mitogen-activated protein kinase 13	1.99	0.00004
HKT0001_H03	Microtubule-associated protein RP/EB	1.99	0.00003
CA363480	Aquaporin-CHIP	1.98	0.00003
EXOB1_H09	ATP synthase beta chain-1	1.96	0.00029
HK0001_D12	Estrogen-responsive B box protein	1.94	0.00153
est02h09	Nonhistone chromosomal protein HMG-17	1.92	0.00003
CA384134	G1/S-specific cyclin D2	1.89	0.00000
HK0003_C02	Collagen alpha 1(I) chain-1	1.89	0.00146
HK0003_D10	Hypothetical-fish 9	1.87	0.00021
EXOB2_G09	Cytochrome c oxidase subunit I-2	1.82	0.00077
EXOB1_H05	Transposase-6	1.82	0.00006
HK0001_A10	Full-length cDNA clone CS0DC006YH13 of Human Neuroblastoma	1.79	0.00410
EXOB1_A06	ADP,ATP carrier protein T2	1.77	0.00000
HK0003_E06	DnaJ homolog subfamily C member 9	1.74	0.00183
EXOB1_H08	ADP,ATP carrier protein 3	1.73	0.00061
EXOB3_A03	SEC13-related protein	1.73	0.00031
EST1-3A_H07	Cytochrome b-1	1.73	0.00398
utu02b11	Collagen a3(I)-2	1.72	0.00004
HK0002_C04	Apolipoprotein E-2	1.72	0.00111
KVkm2_D03	Nuclear protein 1	1.69	0.00197
EXOB3_F10	Nuclease sensitive element binding protein 1-2	1.68	0.00878
EXOB3_G08	Fatty acid-binding protein-1	1.68	0.00051
HK0001_D05	Ubiquitin-like protein SMT3A-2	1.67	0.00017
EXOB1_E12	Serine protease-like protein-3	1.67	0.00095
est01e05	Glutathione S-transferase, mitochondrial	1.64	0.00153
CA363870	Thioredoxin-like protein 4A	1.64	0.00388

Table S3. *Cont.*

Clone ID	Clone Name	FC	*p*-Value
est03d06	Eukaryotic translation initiation factor 2 subunit 2	1.64	0.00211
EST1-3A_G07	COP9 signalosome complex subunit 6	1.63	0.00062
EXOB4_H04	Fructose-1,6-bisphosphatase isozyme 2	1.63	0.00012
est04b01	Similar to rRNA (Vangl2)	1.59	0.00433
HK0002_A12	NADH-ubiquinone oxidoreductase 15 kDa subunit	1.59	0.00417
CA370329	Lysozyme C precursor	1.59	0.00003
EXOB2_H02	Serine/arginine repetitive matrix 1	1.57	0.00020
utu04e11	Hyaluronan and proteoglycan link protein 2 precursor	1.56	0.01000
EXOB2_A04	Hypothetical-fish 4	1.56	0.00371
HK0003_C10	Over-expressed breast tumor protein-like	1.55	0.00396
EXOB4_H08	Alanine-glyoxylate aminotransferase 1	1.55	0.00016
HK0002_B07	Heat shock 70kDa protein 8	1.55	0.00811
EXOB4_E01	Transposase-58	1.54	0.00622
CA361342	Orphan nuclear receptor NR4A2	1.54	0.00093
EXOB2_B05	Hypothetical-fish 27	1.52	0.00301
EXOB2_A02	Nucleoside diphosphate kinase, mitochondrial precursor	1.52	0.00001
HK0002_C02	Chromosome-associated kinesin KIF4A	1.52	0.00332
EXOB2_G01	Leukocyte cell-derived chemotaxin 2	1.51	0.00668
est01e02	Thymosin beta-4-1	1.51	0.00077
est04c05	Ferritin heavy chain-1	1.50	0.00214
est02f12	Proteasome subunit beta type 9 precursor	1.50	0.00022
utu04g05	40S ribosomal protein S9-3	−1.51	0.00015
est01g09	Synapse associated protein	−1.56	0.00000
EXOB2_B01	Nuclease sensitive element binding protein 1-1	−1.57	0.00713
EXOB1_B04	Beta actin-1	−1.58	0.00305
CA388461	NF-kappaB inhibitor alpha-3	−1.59	0.00028
CA362134	T-cell leukemia virus enhancer factor	−1.61	0.00316
CA379787	Cyclin G1	−1.62	0.00095
EXOB3_G05	Actin, cytoplasmic 2	−1.64	0.00383
HK0002_G04	Fructose-bisphosphate aldolase A	−1,66	0.00054
CA366489	Multidrug resistance protein 3-1	−1.67	0.00002
HKT0001_D05	ADP,ATP carrier protein T1	−1.69	0.00154
utu03e09	Nebulin-1	−1.69	0.00068
CA380185	ERO1-like protein alpha precursor	−1.69	0.00249
HK0003_D04	Putative nuclear protein ORF1-FL49	−1.69	0.00002
CA385359	Serum amyloid P-component-2	−1.71	0.00017
Hete0002_A07	Metallothionein-IL	−1.71	0.00150
utu02a10	Glyceraldehyde-3-phosphate dehydrogenase-3	−1.75	0.00000
P_3	Epididymal secretory glutathione peroxidase	−1.77	0.00025
CA362833	Heterogenous nuclear ribonucleoprotein U	−1.79	0.00001
HK0002_B06	Troponin T-3, fast skeletal muscle	−1.80	0.00667
utu03d10	Glyceraldehyde-3-phosphate dehydrogenase-5	−1.84	0.00049
KVkm2_G01	60S ribosomal protein L32-2	−1.88	0.00011
utu03f12	26S proteasome non-ATPase regulatory subunit 14	−1.88	0.00088
utu04d02	Beta enolase-5	−1.90	0.00078

Table S3. *Cont.*

Clone ID	Clone Name	FC	*p*-Value
Hete0002_C10	Creatine kinase, M-1	−1.94	0.00003
utu02c05	Hypothetical-fish 13	−2.00	0.00114
HK0003_H04	NADH-ubiquinone oxidoreductase 19 kDa subunit	−2.00	0.00092
HK0002_E12	Creatine kinase, M-2	−2.03	0.00016
est03b11	Acidic leucine-rich nuclear phosphoprotein 32 A-1	−2.03	0.00169
CR_2	14-3-3 B2	−2.08	0.00157
P_17	Nitric oxide synthase 2 (NOS2)	−2.11	0.00030
utu02e02	Tropomyosin alpha 3 chain-2	−2.12	0.00049
CA363762	Cell death activator CIDE-B	−2.14	0.00000
Hete0002_A06	40S ribosomal protein S11	−2.16	0.00003
KVkm2_E02	Glyceraldehyde-3-phosphate dehydrogenase-2	−2.21	0.00000
utu03h10	Tropomyosin alpha 3 chain-3	−2.26	0.00019
utu03c12	Glyceraldehyde-3-phosphate dehydrogenase-4	−2.28	0.00001
HK0002_F05	Myosin heavy chain, skeletal, fetal	−2.35	0.00007
utu02b01	Apolipoprotein A-IV	−2.36	0.00000
utu03e10	Troponin I-3, fast skeletal muscle	−2.44	0.00001
HK0002_H11	Creatine kinase, B chain	−2.48	0.00033
utu04e07	60S ribosomal protein L39	−2.52	0.00007
utu04c08	Transposase-7	−2.68	0.00001
utu04f01	40S ribosomal protein S9-2	−2.71	0.00001
CR_4	14-3-3 C2	−2.74	0.00002
utu04a06	Creatine kinase, M-3	−2.75	0.00000
KVkm2_E08	Aldehyde dehydrogenase 9 A1-2	−2.80	0.00000
utu03b10	Beta enolase-3	−2.85	0.00000
utu04b04	Hypothetical-fish 32	−2.88	0.00000
HK0003_E07	Myosin light chain 2-1	−2.99	0.00000
utu04f08	Actin, alpha skeletal 5	−3.07	0.00000
utu02f09	Myosin light chain 2-2	−3.21	0.00000
utu03b03	Beta enolase-2	−3.31	0.00000
utu04f06	Myosin heavy chain, skeletal, adult 1-2	−3.32	0.00000
utu04d04	Actin, alpha skeletal 4	−3.39	0.00000
HK0001_B08	Putative pre-mRNA splicing factor RNA helicase	−3.42	0.00000
utu03c08	Beta enolase-4	−3.44	0.00000
utu01g11	Actin, alpha skeletal 3	−3.54	0.00000
utu02b08	High mobility group protein 2	−3.60	0.00000
utu03e08	Sarcoplasmic/endoplasmic reticulum calcium ATPase 2	−3.64	0.00000
HK0001_D01	Ubiquitin	−3.73	0.00000
HK0003_B12	Myosin light chain 1, skeletal muscle isoform	−3.79	0.00000
est03h05	Acidic leucine-rich nuclear phosphoprotein 32 A-2	−3.87	0.00000
utu02c02	Myosin heavy chain, skeletal, adult 1-1	−4.03	0.00000
utu01f04	Actin, alpha skeletal 2	−4.10	0.00000
HK0003_A02	Cation-transporting ATPase	−4.19	0.00000
utu01e06	Troponin I-2, fast skeletal muscle	−4.47	0.00000
HK0003_C11	Tropomyosin alpha 3 chain-1	−4.61	0.00000
utu01f12	Troponin T-1, fast skeletal muscle	−4.61	0.00000

Table S3. *Cont.*

Clone ID	Clone Name	FC	*p*-Value
utu01h05	Troponin T-2, fast skeletal muscle	−4.77	0.00000
utu04a11	Troponin I-4, fast skeletal muscle	−5.55	0.00000
HK0003_G01	Creatine kinase, M-2	−5.76	0.00000
utu04h11	Myosin light chain 2-2	−5.95	0.00000
HK0001_E10	Myosin regulatory light chain 2, ventricular/cardiac muscle isoform	−6.00	0.00000
HK0002_D09	Prothymosin alpha	−6.86	0.00053
HK0003_C08	Parvalbumin alpha-2	−7.94	0.00004
utu02b04	Troponin C-2, skeletal muscle	−8.61	0.00000
HK0002_D05	Parvalbumin alpha-1	−28.74	0.00000
utu03e06	Parvalbumin alpha-3	−74.30	0.00000

> 4	2 to 4	1.5 to 2	−1.5 to −2	−2 to −4	< −4

A SFA 2.0 microarray platform was used for the gene expression analysis in slow skeletal muscle at 72 h after LPS exposure, according to the procedure described in Materials and Methods. Significantly up- and down-regulated genes ($p < 0.01$, Student's test, 12 spot replicates per gene, $-1.5 > FC < -1.5$)) are highlighted with a color scale. Data shown represent mean fold change (FC).

Table S4. Differentially expressed genes in fast skeletal muscle at 24 h after lipopolysaccharide (LPS) administration in rainbow trout.

Clone ID	Clone name	FC	*p*-value
HK0002_D05	Parvalbumin alpha-1	2.33	0.00000
CA347565	Semaphorin sem2	2.04	0.00015
utu03e06	Parvalbumin alpha-3	1.98	0.00000
CA361724	Signal transducer and activator of transcription 3	1.83	0.00002
est01g07	Proteasome activator complex subunit 2	1.83	0.00136
CA381199	94 kDa glucose-regulated protein	1.74	0.00175
CA368961	78 kDa glucose-regulated protein precursor	1.73	0.00492
utu02b11	Collagen a3(I)-2	1.73	0.00003
HK0002_D09	Prothymosin alpha	1.69	0.00000
utu01e09	Embryonic alpha-type globin2+collagen alpha 2(1)	1.64	0.00005
est04e04	MHC class 1b antigen	1.64	0.00382
HKT0001_B03	Alpha 2 type I collagen-1	1.60	0.00000
est02g07	Ig mu heavy chain disease protein	1.58	0.00017
utu04a11	Troponin I-4, fast skeletal muscle	1.58	0.00005
P_55	VEGF6	1.58	0.00376
est04b04	Cytokeratin 8	1.53	0.00023
EXOB2_D06	NADH dehydrogenase subunit 5-1	1.53	0.00272
est03e02	Hypothetical-fish 15	1.52	0.00008
ENH2_F09	Beta-2-microglobulin-1	1.52	0.00103
Hete0002_A07	Metallothionein-IL	−1.50	0.00182
EXOB3_C07	60S ribosomal protein L7a-2	−1.50	0.00702
HKT0001_D05	ADP,ATP carrier protein T1	−1.51	0.00188
EXOB3_F05	Proteasome subunit alpha type 7-1	−1.51	0.00067
HST0001_D02	ATP-binding cassette, sub-family F, member 2	−1.52	0.00321
utu03d10	Glyceraldehyde-3-phosphate dehydrogenase-5	−1.61	0.00001

Table S4. *Cont.*

Clone ID	Clone name	FC	*p*-value
utu03c04	Synaptic glycoprotein SC2-2	−1.61	0.00932
EXOB3_G08	Fatty acid-binding protein-1	−1.64	0.00009
utu01e06	Troponin I-2, fast skeletal muscle	−1.74	0.00001
CA367764	Calmodulin-1	−1.85	0.00030
CA362134	T-cell leukemia virus enhancer factor	−1.85	0.00075
utu03h02	6-phosphofructokinase	−1.88	0.00064
utu02g07	Glycogen phosphorylase-2	−1.98	0.00000
utu02g04	Glycogen phosphorylase-1	−2.11	0.00000
CA382425	B-cell translocation gene 1-2	−2.31	0.00003
EXOB1_H06	CC chemokine SCYA110-2	−2.97	0.00001

> 4	2 to 4	1.5 to 2	−1.5 to −2	−2 to −4	<−4

A SFA 2.0 microarray platform was used for the gene expression analysis in fast skeletal muscle at 24 h after LPS exposure, according to the procedure described in Materials and Methods. Significantly up- and down-regulated genes ($p < 0.01$, Student's test, 12 spot replicates per gene, $-1.5 > FC < -1.5$) are highlighted with a color scale. Data shown represent mean fold change (FC).

Table S5. Differentially expressed genes in fast skeletal muscle at 72 h after lipopolysaccharide (LPS) administration in rainbow trout.

Clone ID	Clone Name	FC	*p*-Value
utu01e09	Embryonic alpha-type globin2+collagen alpha 2(1)	8.07	0.00000
HKT0001_B03	Alpha 2 type I collagen-1	7.48	0.00000
utu04c11	Collagen alpha 1(I) chain-2	7.44	0.00000
HK0003_A11	Apolipoprotein A-I-1	4.57	0.00000
utu02b11	Collagen a3(I)-2	3.85	0.00000
utu04a11	Troponin I-4, fast skeletal muscle	3.76	0.00000
HK0002_D05	Parvalbumin alpha-1	3.65	0.00000
utu02f09	Myosin light chain 2-2	3.20	0.00000
CA371363	Glucose-6-phosphate isomerase-1	3.09	0.00000
utu03e10	Troponin I-3, fast skeletal muscle	3.07	0.00000
EXOB4_B01	Transferrin	2.92	0.00001
CR_8	14-3-3 G2	2.77	0.00000
utu02e02	Tropomyosin alpha 3 chain-2	2.55	0.00000
utu04d02	Beta enolase-5	2.49	0.00000
est03h05	Acidic leucine-rich nuclear phosphoprotein 32 A-2	2.43	0.00000
utu03b10	Beta enolase-3	2.41	0.00000
utu03e06	Parvalbumin alpha-3	2.38	0.00000
utu04f01	40S ribosomal protein S9-2	2.37	0.00005
est04e04	MHC class 1b antigen	2.36	0.00000
utu03h10	Tropomyosin alpha 3 chain-3	2.35	0.00054
utu04f08	Actin, alpha skeletal 5	2.31	0.00009
utu01h05	Troponin T-2, fast skeletal muscle	2.30	0.00000
utu04c08	Transposase-7	2.29	0.00000
HK0001_B08	Putative pre-mRNA splicing factor RNA helicase	2.29	0.00001
est01c08	14-3-3C2	2.28	0.00000

Table S5. *Cont.*

Clone ID	Clone Name	FC	*p*-Value
utu01f12	Troponin T-1, fast skeletal muscle	2.28	0.00000
est04c09	Hypothetical-fish 41	2.24	0.00004
CA370329	Lysozyme C precursor	2.18	0.00008
ENH2_B05	60S ribosomal protein L13a-acute pahse protein	2.14	0.00001
est03b11	Acidic leucine-rich nuclear phosphoprotein 32 A-1	2.11	0.00000
HK0003_C08	Parvalbumin alpha-2	2.11	0.00005
KVkm2_F07	Hepcidin 1	2.11	0.00001
utu04d04	Actin, alpha skeletal 4	2.10	0.00003
CA346925	Acidic leucine-rich nuclear phosphoprotein 32 E	2.10	0.00000
HK0001_E10	Myosin regulatory light chain 2, cardiac	2.09	0.00008
utu03b03	Beta enolase-2	2.09	0.00363
KVkm2_G01	60S ribosomal protein L32-2	2.08	0.00007
CA379787	Cyclin G1	2.07	0.00034
EXOB2_A01	MHC class II invariant chain-like protein 1	2.05	0.00056
Hete0002_A07	Metallothionein-IL	2.04	0.00001
utu03h12	Hypothetical-fish 29	2.03	0.00014
KVkm2_G04	60S ribosomal protein L32-3	2.01	0.00001
utu03c08	Beta enolase-4	2.00	0.00001
HK0002_B06	Troponin T-3, fast skeletal muscle	1.99	0.00004
utu01f04	Actin, alpha skeletal 2	1.96	0.00000
CA366393	Mannan-binding lectin serine protease 2-1	1.95	0.00000
CR_4	14-3-3 C2	1.95	0.00006
utu01e06	Troponin I-2, fast skeletal muscle	1.94	0.00000
CA342760	47 kDa heat shock protein-2	1.90	0.00017
EXOB2_D06	NADH dehydrogenase subunit 5-1	1.89	0.00074
EST1-3A_G02	NADH dehydrogenase subunit 2	1.88	0.00006
est02f12	Proteasome subunit beta type 9 precursor	1.88	0.00015
utu01g11	Actin, alpha skeletal 3	1.87	0.00004
P_17	Nitric oxide synthase 2 (NOS2)	1.83	0.00020
EXOB4_G06	NADH-ubiquinone oxidoreductase B15 subunit	1.82	0.00000
P_3	Epididymal secretory glutathione peroxidase	1.82	0.00002
utu03h03	Nuclear RNA helicase	1.81	0.00478
utu04e07	60S ribosomal protein L39	1.80	0.00064
CA385882	Vesicle-associated membrane protein-associated protein B/C	1.80	0.00000
est02f08	Serine protease-like protein-1	1.78	0.00001
HK0002_D09	Prothymosin alpha	1.78	0.00001
CA362476	DnaJ homolog subfamily B member 6	1.76	0.00014
utu02g04	Glycogen phosphorylase-1	1.76	0.00009
utu04g05	40S ribosomal protein S9-3	1.76	0.00016
HK0003_B10	Pterin-4-alpha-carbinolamine dehydratase	1.76	0.00003
HK0003_A03	Thymosin beta-4-2	1.74	0.00002
utu01g10	Keratin, type II cytoskeletal 8	1.74	0.00000
utu03f07	NADH dehydrogenase subunit 5-2	1.74	0.00000
utu03e09	Nebulin-1	1.74	0.00017

Table S5. *Cont.*

Clone ID	Clone Name	FC	*p*-Value
est01e09	Seryl-tRNA synthetase	1.73	0.00003
CA384986	Thyrotropin-releasing hormone degrading ectoenzyme	1.72	0.00019
P_46	ATPase 6	1.72	0.00035
est04c07	Ferritin heavy chain-2	1.70	0.00012
CA386226	Cyclophilin-like 6	1.69	0.00001
utu03d10	Glyceraldehyde-3-phosphate dehydrogenase-5	1.66	0.00296
utu03g10	Cathepsin D	1.66	0.00007
EXOB3_E07	F-actin capping protein alpha-1 subunit	1.65	0.00060
CR_7	14-3-3 G1	1.64	0.00019
utu04b09	Complement factor H-3	1.63	0.00007
HK0003_E07	Myosin light chain 2-1	1.63	0.00002
HK0003_B12	Myosin light chain 1, skeletal muscle isoform	1.63	0.00976
HK0003_D04	Putative nuclear protein ORF1-FL49	1.63	0.00000
est04c05	Ferritin heavy chain-1	1.63	0.00001
est04e06	Caspase recruitment domain protein 4	1.61	0.00011
CA385591	DnaJ homolog subfamily B member 11 precursor	1.61	0.00022
CA376350	Serum amyloid P-component-1	1.60	0.00155
HK0003_H04	NADH-ubiquinone oxidoreductase 19 kDa subunit	1.60	0.00034
utu02a06	Collagen a3(I)-1	1.58	0.00234
CA342145	RNA binding motif protein 4	1.57	0.00047
CR_6	14-3-3 E2	1.57	0.00100
ENH2_F09	Beta-2-microglobulin-1	1.56	0.00002
CR_2	14-3-3 B2	1.56	0.00000
CA363762	Cell death activator CIDE-B	1.56	0.00041
HKT0001_H03	Microtubule-associated protein RP/EB	1.55	0.00052
CA367764	Calmodulin-1	1.55	0.00126
P_8	Glutathione reductase, mitochondrial-1	1.54	0.00000
utu03f08	Nebulin-2	1.54	0.00837
utu02g07	Glycogen phosphorylase-2	1.51	0.00009
EST1-3A_B06	Ring finger protein 10	−1.51	0.00177
EXOB3_D08	Histone H33-2	−1.51	0.00064
utu04f06	Myosin heavy chain, skeletal, adult 1-2	−1.51	0.00825
CA352520	D-type cyclin-interacting protein 1	−1.52	0.00013
EXOB3_G03	60S acidic ribosomal protein P2	−1.53	0.00000
CA357173	Peptidyl-prolyl cis-trans isomerase 2-2	−1.53	0.00773
utu01e12	Zinc finger protein 228	−1.53	0.00054
est03g06	NSFL1 cofactor p47	−1.54	0.00168
est04b01	Similar to rRNA (Vangl2)	−1.54	0.00010
CA376978	CD2 binding protein 1-2	−1.54	0.00054
EST1-3A_E05	Hpa repeat-2	−1.55	0.00001
EST1-3A_F12	Transposase-59	−1.56	0.00621
CA384452	Heat shock protein HSP 90-alpha	−1.56	0.00504
CA366403	Heat shock 27 kDa protein-1	−1.56	0.00000
EXOB1_C10	Cytochrome P450 2K4-2	−1.57	0.00003

Table S5. *Cont.*

Clone ID	Clone Name	FC	*p*-Value
CA381625	Zinc finger protein 148	−1.58	0.00020
EXOB1_D11	Dynein light chain 2, cytoplasmic	−1.58	0.00063
CA373890	Heat shock protein HSP 90-beta-1	−1.59	0.00001
EXOB4_B09	Macrophage receptor MARCO	−1.59	0.00663
utu03g04	Myosin heavy chain, cardiac muscle beta isoform	−1.61	0.00086
CA378361	Ubiquitin ligase SIAH1	−1.62	0.00010
est02b03	Beta-ureidopropionase	−1.68	0.00012
EXOB2_D11	E3 ligase for inhibin receptor	−1.69	0.00025
EST1-3A_H07	Cytochrome b-1	−1.70	0.00310
EXOB4_C12	Gastrulation specific protein G12	−1.74	0.00234
CA368739	BCL2-associated athanogene 1	−1.74	0.00732
HK0002_B10	B-cell receptor-associated protein BAP37-2	−1.77	0.00018
EXOB2_B04	Syntenin 1	−1.79	0.00020
CA371300	Acid ceramidase precursor	−1.79	0.00053
EST1-3A_H05	Adenosine deaminase 3	−1.80	0.00249
CA364424	Cyclophilin-40	−1.81	0.00003
CA365039	CD63	−1.81	0.00001
EXOB3_H04	Transposase-1	−1.82	0.00003
CA358990	B-cell lymphoma 6 protein-2	−1.83	0.00003
EXOB1_F02	Transcription regulator protein BACH1	−1.85	0.00057
CR_5	14-3-3 E1	−1.92	0.00000
EXOB2_F08	CDC10 protein homolog	−1.97	0.00004
CA378114	Thioredoxin-like protein 1	−1.98	0.00334
EXOB1_H05	Transposase-6	−1.98	0.00002
EST1-3A_H06	Transcription factor jun-B-1	−2.00	0.00001
est01c06	ADP-ribosylation factor 4	−2.00	0.00009
EXOB2_F01	Proteasome subunit alpha type 6	−2.01	0.00073
EXOB2_G10	Histone H33-1	−2.01	0.00001
CA382570	Mitogen-activated protein kinase 13	−2.07	0.00000
P_56	RIB300	−2.08	0.00006
CA367914	Forkhead box protein O3A	−2.10	0.00010
EXOB4_D12	Hpa repeat-1	−2.11	0.00000
EXOB2_B07	A+U-rich element RNA binding factor	−2.15	0.00004
EXOB3_G08	Fatty acid-binding protein-1	−2.18	0.00000
utu02a12	Brain protein 44-like protein	−2.22	0.00011
CA344228	Phospholipase A-2-activating protein	−2.25	0.00001
CA348284	CCAAT/enhancer binding protein beta	−2.27	0.00121
est04f01	Polyposis locus protein 1	−2.28	0.00045
EXOB2_D01	Proteasome subunit alpha type 3	−2.39	0.00033
EXOB2_F07	T-cell lymphoma associated antigen se33-1	−2.47	0.00000
CA361724	Signal transducer and activator of transcription 3	−2.56	0.00009
EXOB1_H08	ADP,ATP carrier protein 3	−2.66	0.00000
utu02a08	Ubiquitin and ribosomal protein S27a-2	−2.67	0.00000
CA368052	Myosin-like BCL2-interacting protein	−2.84	0.00000
est03a08	Cytochrome c-1	−3.08	0.00000

Table S5. *Cont.*

Clone ID	Clone Name	FC	*p*-Value
CA349860	ATP-dependent Clp protease ATP-binding subunit	−3.37	0.00333
CA363848	p53-regulated protein PA26	−3,39	0,00000
KVkm2_H06	Hypothetical-fish 22	−3,49	0,00000
CA379751	Electron transfer flavoprotein-ubiquinone oxidoreductase	−3,50	0,00000
KVkm2_G02	Proteasome subunit alpha type 7-2	−3,85	0,00000
est01g09	Synapse associated protein	−4,50	0,00000
EXOB3_F05	Proteasome subunit alpha type 7-1	−4,52	0,00000
utu03e01	26S protease regulatory subunit 6B-2	−4,67	0,00000
CA388461	NF-kappaB inhibitor alpha-3	−4,68	0,00000
utu03e11	Troponin I, slow skeletal muscle	−4,88	0,00000
CA377007	Growth arrest and DNA-damage-inducible GADD45 gamma-1	−4,91	0,00007
est04f07	Transposase-5	−5,05	0,00000
est04h08	ADP-ribosylation factor 3	−5,35	0,00000
utu03c11	26S protease regulatory subunit 6B-1	−5,35	0,00000
CA372952	Survival of motor neuron-related splicing factor 30	−5,35	0,00000
CA377838	Mitogen activated protein kinase activated protein kinase-3	−6,31	0,00000
HK0002_G03	Histone H10	−6,49	0,00000
CA382425	B-cell translocation gene 1-2	−6,76	0,00000
utu03f12	26S proteasome non-ATPase regulatory subunit 14	−7,06	0,00000
CA380011	Cullin homolog 1	−9,36	0,00000
HK0001_D01	Ubiquitin	−265,15	0,00000

> 4	2 to 4	1.5 to 2	−1.5 to −2	−2 to −4	< −4

A SFA 2.0 microarray platform was used for the gene expression analysis in fast skeletal muscle at 72 h after LPS exposure, according to the procedure described in Materials and Methods. Significantly up- and down-regulated genes ($p < 0.01$, Student's test, 12 spot replicates per gene, −1,5 > FC < −1,5)) are highlighted with a color scale. Data shown represent mean fold change (FC).

Table S6. Differentially expressed genes that are common to slow and fast muscles at 24 h after LPS administration ($p < 0.01$)

		24 h	
**Clone ID**	**Clone Name**	**Fast Muscle**	**Slow Muscle**
utu03h02	6-phosphofructokinase	−1.88	−1.53
CA368961	78 kDa glucose-regulated protein precursor	1.73	1.72
HKT0001_B03	Alpha 2 type I collagen-1	1.60	2.16
ENH2_F09	Beta-2-microglobulin-1	1.52	2.13
EXOB1_H06	CC chemokine SCYA110-2	−2.97	−2.14
utu02b11	Collagen a3(I)-2	1.73	2.11
est04b04	Cytokeratin 8	1.53	1.51
utu01e09	Embryonic alpha-type globin2+collagen alpha 2(1)	1.64	1.63
EXOB3_G08	Fatty acid-binding protein-1	−1.64	−1.63
est04e04	MHC class 1b antigen	1.64	2.45
utu03e06	Parvalbumin alpha-3	1.98	1.81
est01g07	Proteasome activator complex subunit 2	1.83	1.60
utu01e06	Troponin I-2, fast skeletal muscle	−1.74	2.43
utu04a11	Troponin I-4, fast skeletal muscle	1.58	1.72

> 4	2 to 4	1.5 to 2	−1.5 to −2	−2 to −4	< −4

A SFA 2.0 microarray platform was used for the gene expression analysis in slow and fast skeletal muscle at 24 h after LPS exposure, according to the procedure described in Materials and Methods. Significantly up- and down-regulated genes ($p < 0.01$, Student's test, 12 spot replicates per gene, −1,5 > FC < −1,5)) are highlighted with a color scale. Data shown represent mean fold change (FC).

Table S7. Differentially expressed genes that are common to slow and fast muscles at 72 h after LPS administration ($p < 0.01$).

Clone ID	Clone Name	72 h	
		Fast Muscle	Slow Muscle
CR_2	14-3-3 B2	1.56	−2.08
CR_4	14-3-3 C2	1.95	−2.74
utu03f12	26S proteasome non-ATPase regulatory subunit 14	−7.06	−1.88
utu04f01	40S ribosomal protein S9-2	2.37	−2.71
utu04g05	40S ribosomal protein S9-3	1.76	−1.51
KVkm2_G01	60S ribosomal protein L32-2	2.08	−1.88
utu04e07	60S ribosomal protein L39	1.80	−2.52
est03b11	Acidic leucine-rich nuclear phosphoprotein 32 A-1	2.11	−2.03
est03h05	Acidic leucine-rich nuclear phosphoprotein 32 A-2	2.43	−3.87
utu01f04	Actin, alpha skeletal 2	1.96	−4.10
utu01g11	Actin, alpha skeletal 3	1.87	−3.54
utu04d04	Actin, alpha skeletal 4	2.10	−3.39
utu04f08	Actin, alpha skeletal 5	2.31	−3.07
EXOB1_H08	ADP,ATP carrier protein 3	−2.66	1.73
HKT0001_B03	Alpha 2 type I collagen-1	7.48	3.53
P_46	ATPase 6	1.72	3.40
utu03b03	Beta enolase-2	2.09	−3.31
utu03b10	Beta enolase-3	2.41	−2.85
utu03c08	Beta enolase-4	2.00	−3.44
utu04d02	Beta enolase-5	2.49	−1.90
CA363762	Cell death activator CIDE-B	1.56	−2.14
utu02a06	Collagen a3(I)-1	1.58	2.93
utu02b11	Collagen a3(I)-2	3.85	1.72
utu04c11	Collagen alpha 1(I) chain-2	7.44	2.81
CA379787	Cyclin G1	2.07	−1.62
EST1-3A_H07	Cytochrome b-1	−1.70	1.73
utu01e09	Embryonic alpha-type globin2+collagen alpha 2(1)	8.07	3.70
P_3	Epididymal secretory glutathione peroxidase	1.82	−1.77
EXOB3_G08	Fatty acid-binding protein-1	−2.18	1.68
est04c05	Ferritin heavy chain-1	1.63	1.50
utu03d10	Glyceraldehyde-3-phosphate dehydrogenase-5	1.66	−1.84
CA370329	Lysozyme C precursor	2.18	1.59
Hete0002_A07	Metallothionein-IL	2.04	−1.71
EXOB2_A01	MHC class II invariant chain-like protein 1	2.05	2.19
HKT0001_H03	Microtubule-associated protein RP/EB	1.55	1.99
CA382570	Mitogen-activated protein kinase 13	−2.07	1.99
utu03g04	Myosin heavy chain, cardiac muscle beta isoform	−1.61	2.00
utu04f06	Myosin heavy chain, skeletal, adult 1-2	−1.51	−3.32
HK0003_B12	Myosin light chain 1, skeletal muscle isoform	1.63	−3.79
HK0003_E07	Myosin light chain 2-1	1.63	−2.99
utu02f09	Myosin light chain 2-2	3.20	−3.21
HK0001_E10	Myosin regulatory light chain 2, cardiac isoform	2.09	−6.00

Table S7. *Cont.*

Clone ID	Clone Name	72 h	
		**Fast Muscle**	**Slow Muscle**
EST1-3A_G02	NADH dehydrogenase subunit 2	1.88	3.06
EXOB2_D06	NADH dehydrogenase subunit 5-1	1.89	7.22
utu03f07	NADH dehydrogenase subunit 5-2	1.74	3.78
HK0003_H04	NADH-ubiquinone oxidoreductase 19 kDa subunit	1.60	−2.00
utu03e09	Nebulin-1	1.74	−1.69
CA388461	NF-kappaB inhibitor alpha-3	−4.68	−1.59
P_17	Nitric oxide synthase 2 (NOS2)	1.83	−2.11
HK0002_D05	Parvalbumin alpha-1	3.65	−28.74
HK0003_C08	Parvalbumin alpha-2	2.11	−7.94
utu03e06	Parvalbumin alpha-3	2.38	−74.30
est02f12	Proteasome subunit beta type 9 precursor	1.88	1.50
HK0002_D09	Prothymosin alpha	1.78	−6.86
HK0003_D04	Putative nuclear protein ORF1-FL49	1.63	−1.69
HK0001_B08	Putative pre-mRNA splicing factor RNA helicase	2.29	−3.42
est02f08	Serine protease-like protein-1	1.78	2.24
est04b01	Similar to rRNA (Vangl2)	−1.54	1.59
est01g09	Synapse associated protein	−4.50	−1.56
HK0003_A03	Thymosin beta-4-2	1.74	2.60
EXOB1_H05	Transposase-6	−1.98	1.82
utu04c08	Transposase-7	2.29	−2.68
utu02e02	Tropomyosin alpha 3 chain-2	2.55	−2.12
utu03h10	Tropomyosin alpha 3 chain-3	2.35	−2.26
utu01e06	Troponin I-2, fast skeletal muscle	1.94	−4.47
utu03e10	Troponin I-3, fast skeletal muscle	3.07	−2.44
utu04a11	Troponin I-4, fast skeletal muscle	3.76	−5.55
utu01f12	Troponin T-1, fast skeletal muscle	2.28	−4.61
utu01h05	Troponin T-2, fast skeletal muscle	2.30	−4.77
HK0002_B06	Troponin T-3, fast skeletal muscle	1.99	−1.80
HK0001_D01	Ubiquitin	−265.15	−3.73

> 4	2 to 4	1.5 to 2	−1.5 to −2	−2 to −4	< −4

A SFA 2.0 microarray platform was used for the gene expression analysis in slow and fast skeletal muscle at 72 h after LPS exposure, according to the procedure described in Materials and Methods. Significantly up- and down-regulated genes ($p < 0.01$, Student's test, 12 spot replicates per gene, −1.5 > FC < −1.5) are highlighted with a color scale. Data shown represent mean fold change (FC).

© 2015 by the authors; licensee MDPI, Basel, Switzerland. This article is an open access article distributed under the terms and conditions of the Creative Commons Attribution license (http://creativecommons.org/licenses/by/4.0/).
